# Supplementary material for: Electrocatalytic hydrogenation of quinolines with water over a fluorine-modified cobalt catalyst
Source: Nat Commun. 2022 Sep 8;13:5297. doi: 10.1038/s41467-022-32933-6 (PMC9458668; doi:10.1038/s41467-022-32933-6)
Supplement: Supplementary file 1 — Supplementary Information [file 41467_2022_32933_MOESM1_ESM.pdf]

Supplementary Information

Electrocatalytic hydrogenation of quinoline with water over a fluorine-modified cobalt catalyst

Guo et al.

Contents

Supplementary Figure 1. Theoretical adsorption configurations of quinoline on the Co(111) and Co(111)-F surfaces.

Supplementary Figure 2. SEM, TEM, HRTEM, and elemental mapping images of Co(OH)F NWs.

Supplementary Figure 3. Synthesis of Co-F NWs by electroreduction of Co(OH)F NWs.

Supplementary Figure 4. Experimental and simulated XAFS spectra of Co-F at the Co K-edge.

Supplementary Figure 5. XRD patterns and EDS results of Co(OH)F NWs and Co-F NWs.

Supplementary Figure 6. SEM, TEM, HRTEM images and elemental mapping of Co-F NWs.

Supplementary Figure 7. Reaction setup.

Supplementary Figure 8. Performance studies and contrast experiments.

Supplementary Figure 9. The correct calibration curves with an internal standard dodecane.

Supplementary Figure 10. Electrocatalytic hydrogenation of **1a** over a nickel foam cathode at different applied potentials.

Supplementary Figure 11. Time-dependent **1a** conversion and **2a** selectivity over Co-F and Co(OH)F within 3 h, respectively.

Supplementary Figure 12. Cycle-dependent conversions of **1a** over Co-F at −1.1 V vs. Hg/HgO within 6h.

Supplementary Figure 13. XPS spectra of the Co-F before and after recyclability test.

Supplementary Figure 14. EIS plots and double-layer capacitance of Co(OH)F NWs and Co-F NWs.

Supplementary Figure 15. Deuterated experiment.

Supplementary Figure 16. The effect of cations in the electrolyte on the hydrogenation of **1a** over Co foil.

Supplementary Figure 17. The effect of pH on the hydrogenation of **1a** over Co-F.

Supplementary Figure 18. Electrocatalytic hydrogenation of **1a** over the 1-dodecanethiol modified Co-F cathode.

Supplementary Figure 19. Electrocatalytic hydrogenation of **1a** in anhydrous CH_3CN in the absence and presence of H_2 over Co-F.

Supplementary Figure 20. Proposed reaction mechanism.

Supplementary Figure 21. Performance of Co foil with and without adding NaF for other electrocatalytic hydrogenation reactions.

Supplementary Figure 22. A qualitative analysis of possible products by GC-MS for electrocatalytic hydrogenation of **6-ethynylquinoline**.

Supplementary Figure 23. A qualitative analysis of possible products by GC-MS for electrocatalytic hydrogenation of **quinoline-6-carbonitrile**.

Supplementary Figure 24. A qualitative analysis of possible products by GC-MS for electrocatalytic hydrogenation of **quinoline-6-carbaldehyde**.

Supplementary Figure 25. Electrocatalytic hydrogenation of **1a** with H_2O over Co-F at a constant current density of −100 mA cm^{-2}.

Supplementary Table 1. EXAFS fitting parameters of a Co-F catalyst.

Supplementary Table 2. pH values of different electrolytes (6.0 mL) with and without adding dioxane (1.0 mL).

Supplementary Table 3. Comparisons of the hydrogenation of quinolines to 1,2,3,4-tetrahydroquinolines between representative heterogeneous catalytic methods over non-noble metal catalysts and our method over Co-F.

Supplementary Notes 1-9.

Supplementary Figures 26-46. NMR spectra of **2a-2q**.

GC-MS spectra of 2r-2s and 5a.

References (1-15).

Supplementary Figures

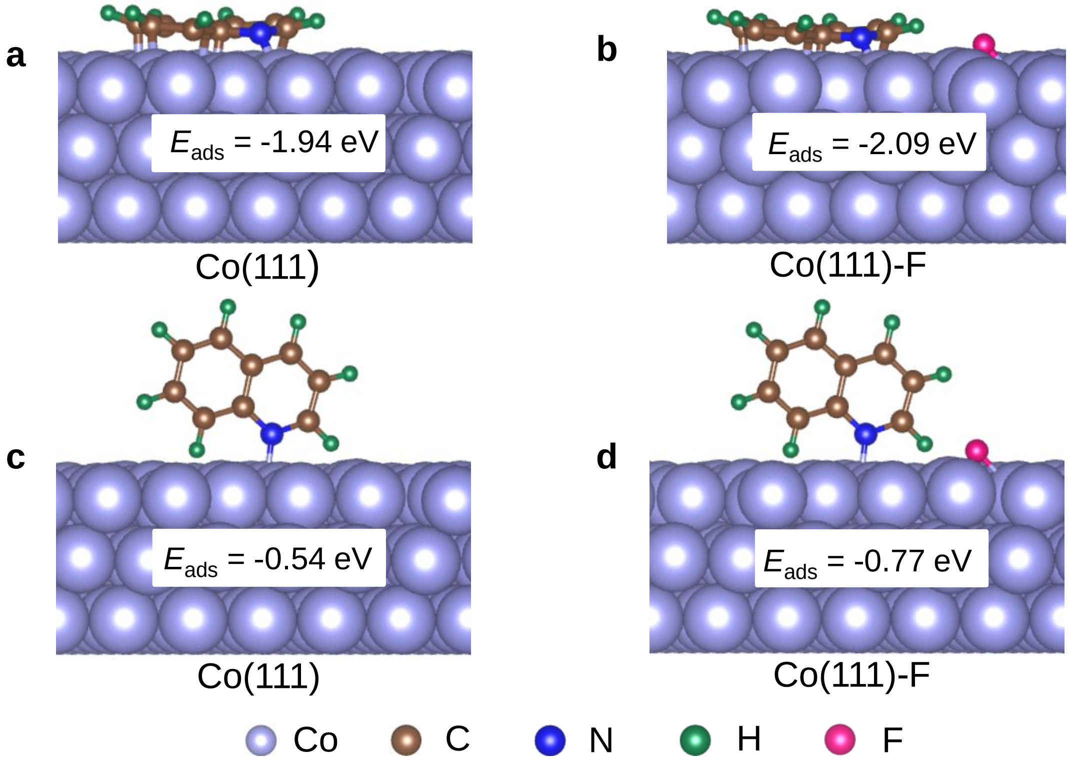

Supplementary Figure 1. Theoretical adsorption configurations of quinoline on the Co(111) and Co(111)-F surfaces. **a** and **b** flat adsorption. **c** and **d** vertical adsorption.

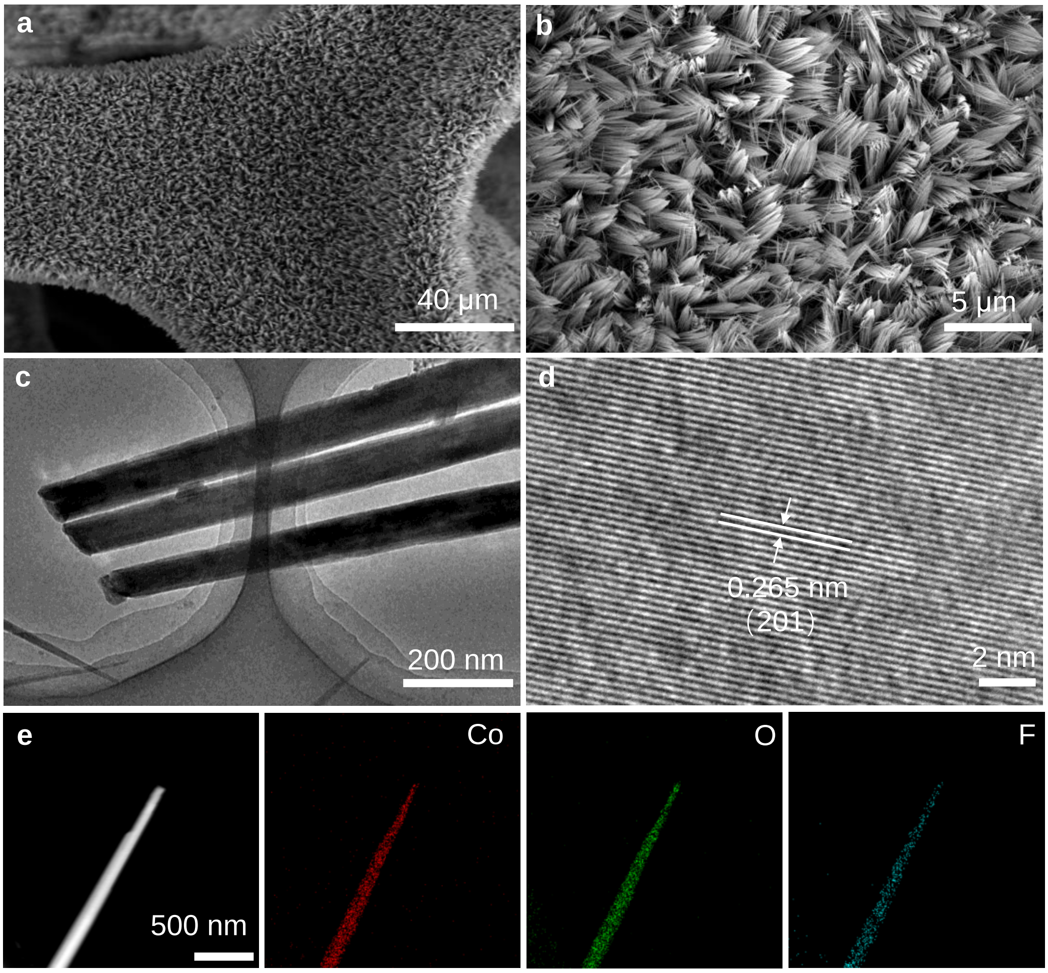

Supplementary Figure 2. SEM, TEM, HRTEM, and elemental mapping images of Co(OH)F NWs. **a** and **b** SEM images, **c** TEM images, **d** HRTEM images, and **e** HAADF images and elemental mapping.

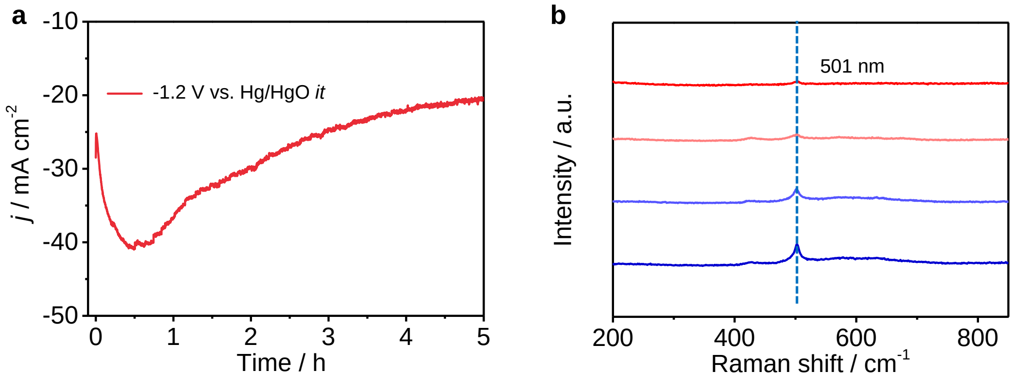

Supplementary Figure 3. Synthesis of Co-F NWs by electroreduction of Co(OH)F NWs. a Chronoamperometry at a constant potential of −1.2 V vs. Hg/HgO in 1.0 M KOH electrolyte. **b** In situ Raman spectra of Co(OH)F NWs at −1.2 V vs. Hg/HgO in 1.0 M KOH electrolyte.

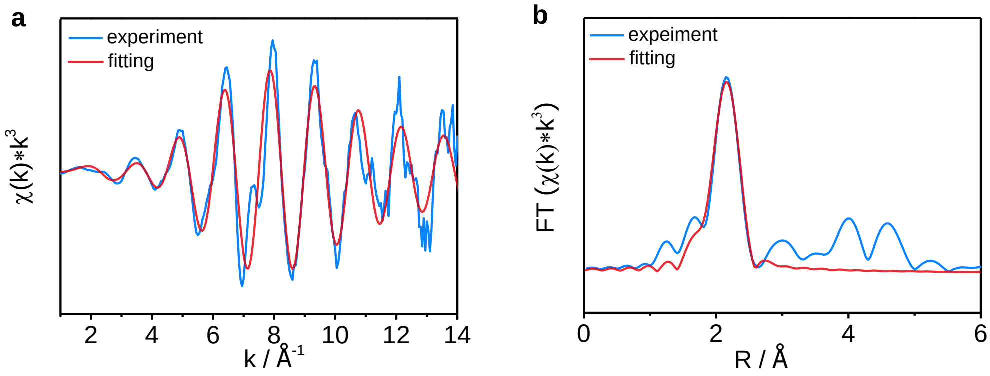

Supplementary Figure 4. Experimental and simulated XAFS spectra of Co-F at the Co K-edge. a The χ(k) data weighted by k^3 and **b** Fourier transformed (FT) to R-space (the k-space ranging from 2 to 14 $Å^{-1}$) to isolate the EXAFS contributions from each coordination shell. Quantified fitting results are shown in Supplementary Table 1.

a

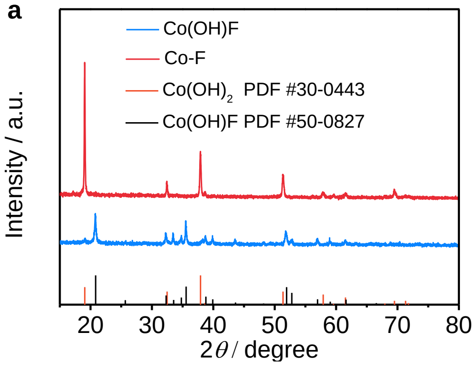

b

Element	Co	O	F
Atomic %	32.22	34.69	33.09

c

Element	Co	O	F
Atomic %	49.30	44.62	6.08

Supplementary Figure 5. XRD patterns and EDS results of Co(OH)F NWs and Co-F NWs. a XRD patterns of Co(OH)F NWs and Co-F NWs. **b** EDS results of Co(OH)F NWs. **c** EDS results of Co-F NWs.

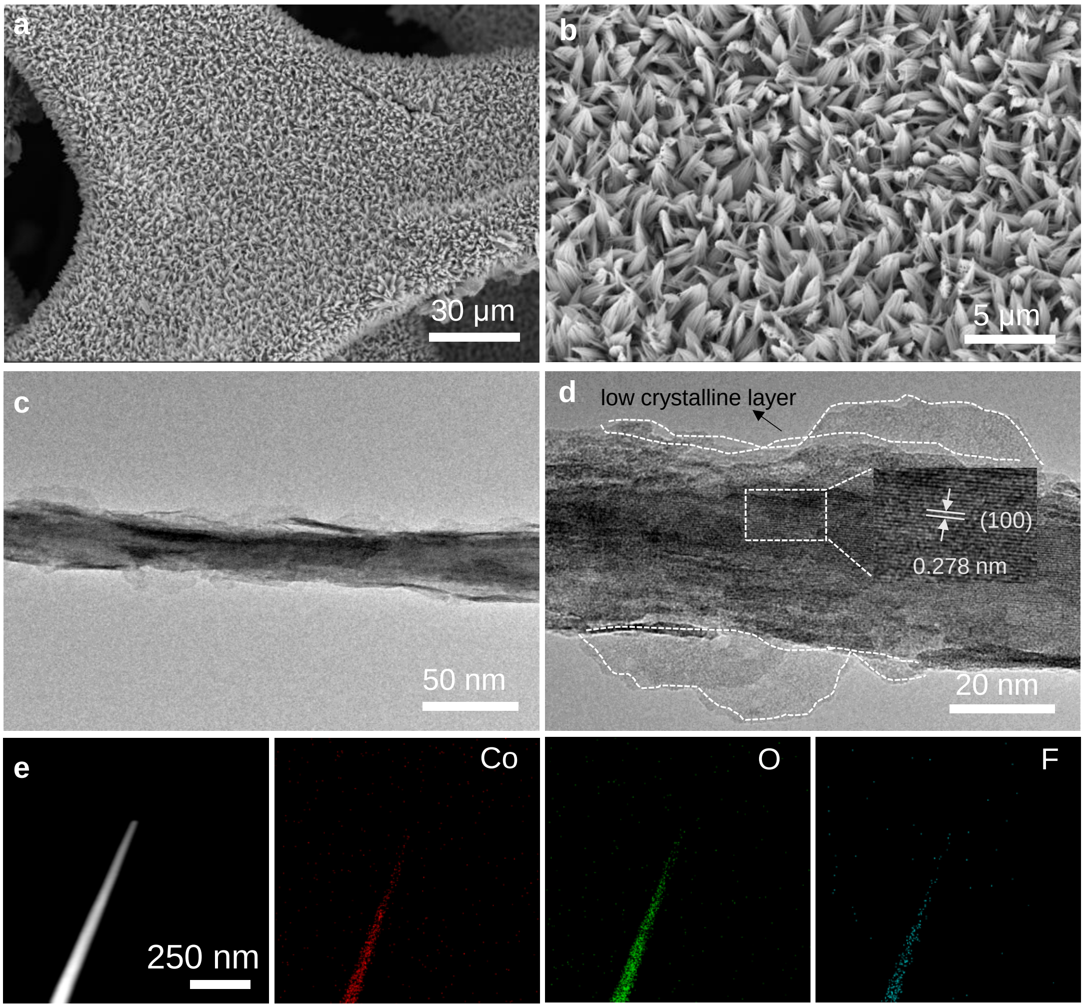

Supplementary Figure 6. SEM, TEM, HRTEM images and elemental mapping of Co-F NWs. a and **b** SEM images, **c** TEM images, **d** HRTEM images, and **e** HAADF images and elemental mapping.

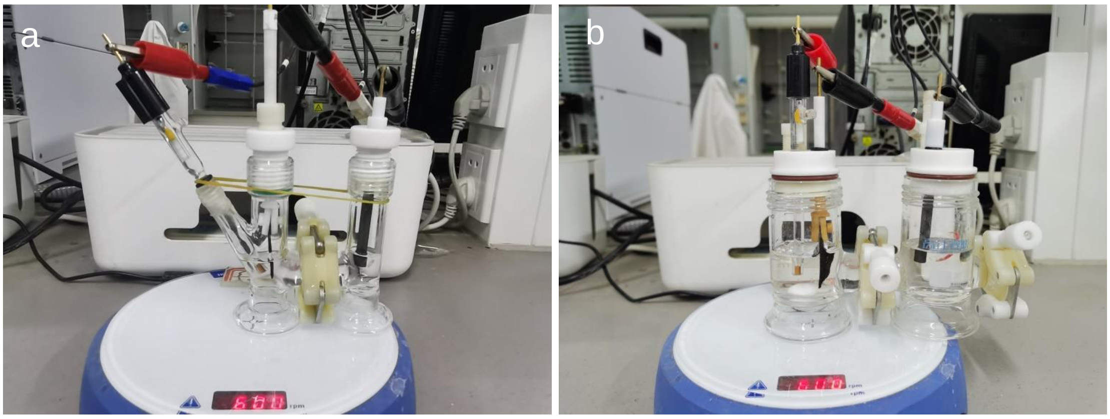

Supplementary Figure 7. Reaction setup. a Reaction setup for electrocatalytic hydrogenation (deuteration) of quinolines. **b** Reaction setup for gram-scale electrocatalytic hydrogenation of **1a** and **1b**.

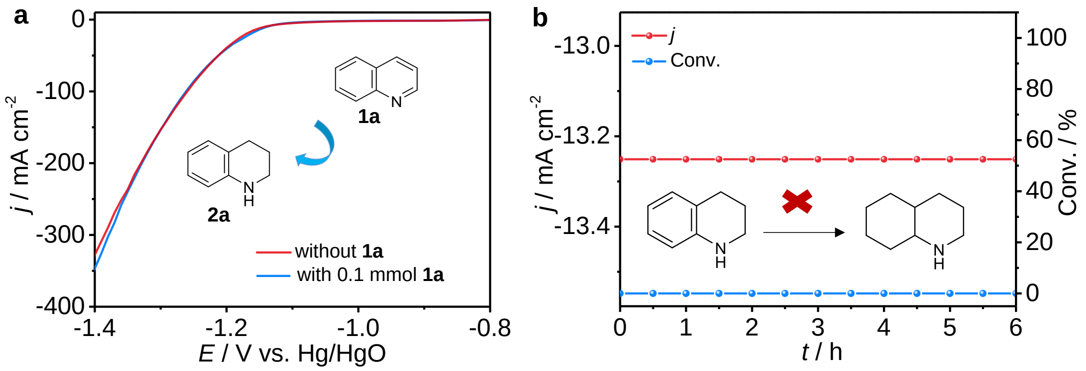

Supplementary Figure 8. Performance studies and contrast experiments. a LSV curves of Co-F cathode at a scan rate of 10 mV s^{-1} in a mixed solution of 1.0 M KOH/dioxane (6:1 v/v, 7 mL) with and without 0.1 mmol of **1a**. **b** Time-dependent tetrahydroquinoline (**2a**) conversion (Conv.) and current (*j*) variations over Co-F. Reaction conditions: **2a** (0.1 mmol, 14.28 mmol L^{-1}), Co-F (working area: 1.0 cm^2), a mixed solution of 1.0 M KOH/dioxane (6:1 v/v, 7 mL), −1.1 V vs. Hg/HgO, RT, 6 h.

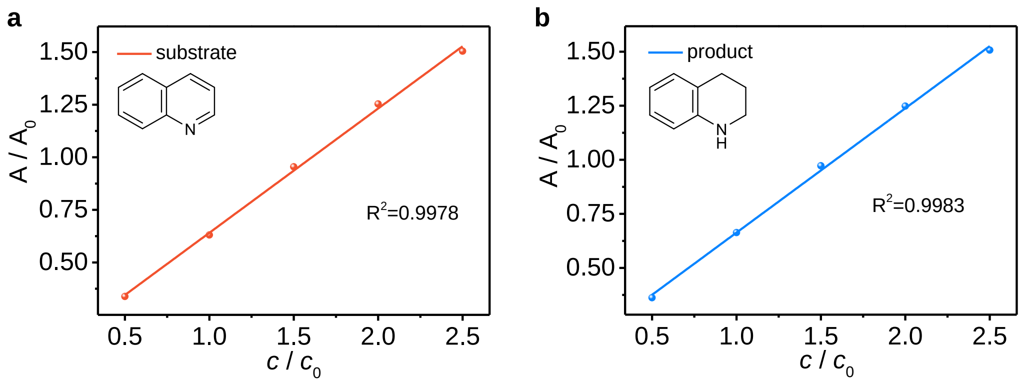

Supplementary Figure 9. The correct calibration curves with an internal standard dodecane. a Quantitative analysis of quinoline (**1a**). **b** Quantitative analysis of 1,2,3,4-tetrahydroquinoline (**2a**).

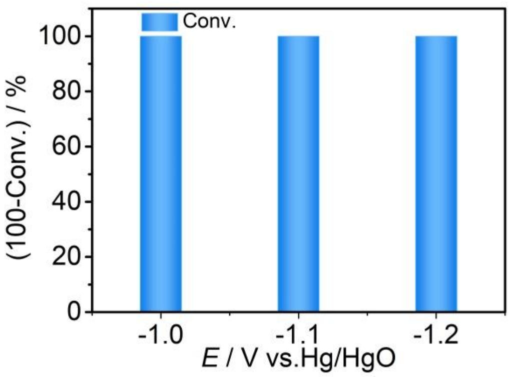

Supplementary Figure 10. Electrocatalytic hydrogenation of 1a over a nickel foam cathode at different applied potentials. Reaction conditions: **1a** (0.1 mmol, 14.28 mmol L^{-1}), NF (working area: 1.0 cm^2), a mixed solution of 1.0 M KOH/dioxane (6:1 v/v, 7 mL), different potentials, RT, 6 h.

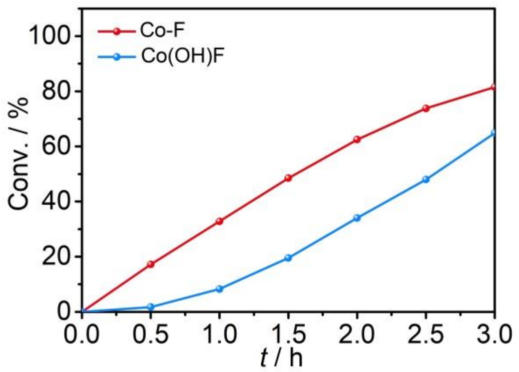

Supplementary Figure 11. Time-dependent 1a conversion and 2a selectivity over Co-F and Co(OH)F within 3 h, respectively. Reaction conditions: **1a** (0.1 mmol, 14.28 mmol L^{-1}), Co-F and Co(OH)F (working area: 1.0 cm^2), a mixed solution of 1.0 M KOH/dioxane (6:1 v/v, 7 mL), −1.1 V vs. Hg/HgO, RT, 3 h.

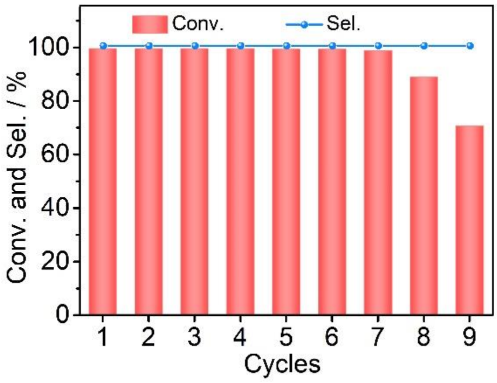

Supplementary Figure 12. Cycle-dependent conversions of 1a over Co-F at −1.1 V vs. Hg/HgO within 6h. Reaction conditions: **1a** (0.1 mmol, 14.28 mmol L^{-1}), Co-F (working area: 1.0 cm^2), a mixed solution of 1.0 M KOH/dioxane (6:1 v/v, 7 mL), −1.1 V vs. Hg/HgO, RT, 6 h.

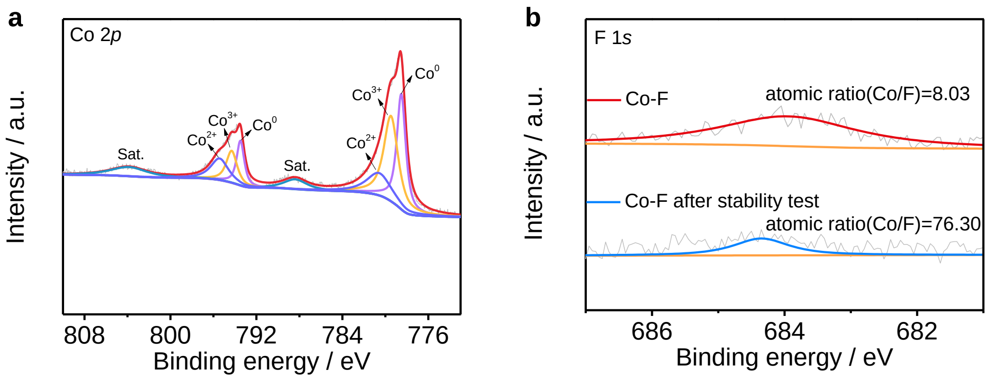

Supplementary Figure 13. XPS spectra of the Co-F before and after recyclability test. a Co 2*p* XPS spectrum of the Co-F. **b** F 1*s* XPS spectrum of the Co-F before and after recyclability test.

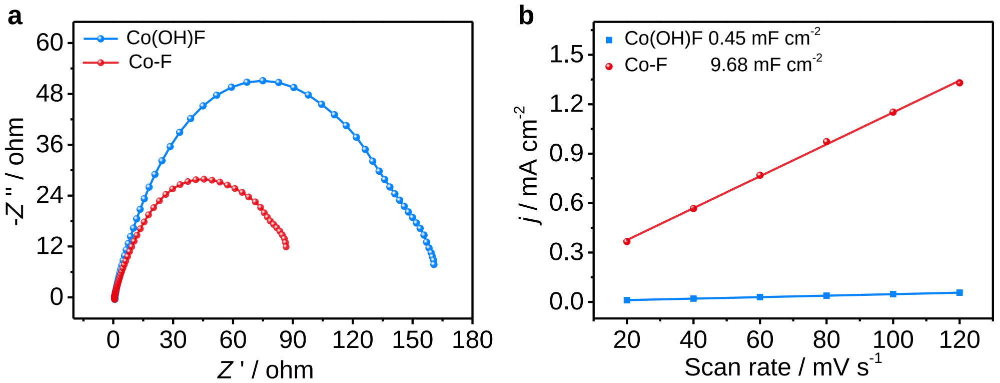

Supplementary Figure 14. EIS plots and double-layer capacitance of Co(OH)F NWs and Co-F NWs. a EIS plots of Co(OH)F NWs and Co-F NWs in a three-electrode system. **b** Double-layer capacitance of Co(OH)F NWs and Co-F NWs.

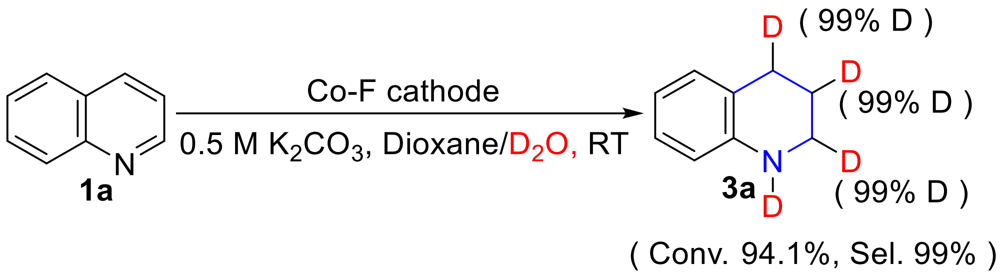

Supplementary Figure 15. Deuterated experiment. Reaction conditions: **1a** (0.2 mmol, 28.57 mmol L^{-1}), Co-F (working area: 1.0 cm^2), a mixed solution of 0.5 M K_2CO_3 (D_2O)/dioxane (6:1 v/v, 7 mL), −1.1 V vs. Hg/HgO, RT, 16 h.

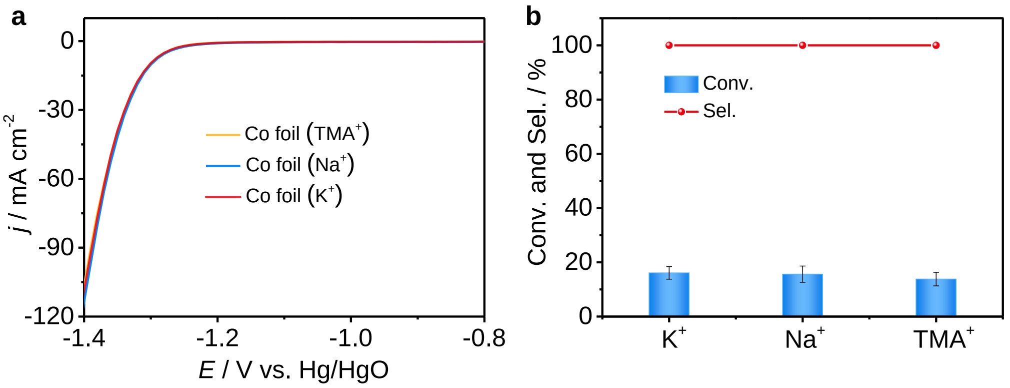

Supplementary Figure 16. The effect of cations in the electrolyte on the hydrogenation of 1a over Co foil. a LSV curves of Co foil recorded in 1.0 M MOH (M = TMA^+, Na^+, and K^+) without **1a** at a scan rate of 10 mV s^{-1}. **b 1a** Conv. and **2a** Sel. obtained over Co foil. Reaction conditions: **1a** (0.1 mmol, 14.28 mmol L^{-1}), Co foil (working area: 1.0 cm^2), a mixed solution of 1.0 M MOH (M = TMA^+, Na^+, and K^+)/dioxane (6:1 v/v, 7 mL), −1.1 V vs. Hg/HgO, RT, 6 h. Error bars correspond to the standard deviation of three independent measurements.

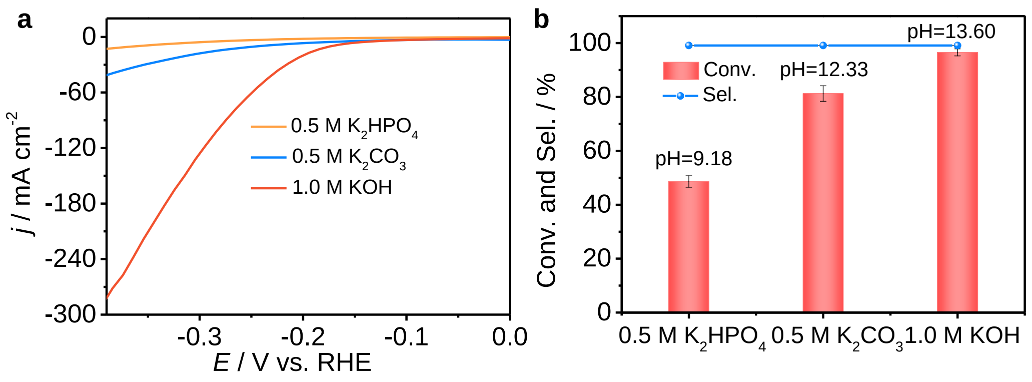

Supplementary Figure 17. The effect of pH on the hydrogenation of 1a over Co-F. **a** LSV curves of Co-F cathode at a scan rate of 10 mV s^{-1} without **1a** in 1.0 M KOH, 0.5 M K_2CO_3, and 0.5 M K_2HPO_4, respectively. **b** **1a** Conv. and **2a** Sel. obtained in the mixed solution of dioxane with 1.0 M KOH, 0.5 M K_2CO_3, and 0.5 M K_2HPO_4, respectively. Reaction conditions: **1a** (0.1 mmol, 14.28 mmol L^{-1}), Co-F (working area: 1.0 cm^2), a mixed solution of different electrolytes/dioxane (6:1 v/v, 7 mL), −0.2 V vs. RHE, RT, 6 h. Error bars correspond to the standard deviation of three independent measurements.

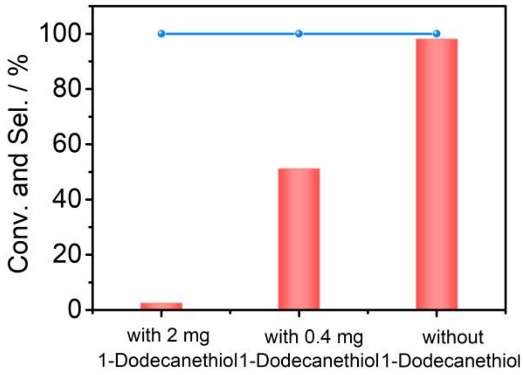

Supplementary Figure 18. Electrocatalytic hydrogenation of 1a over the 1-dodecanethiol modified Co-F cathode. Reaction conditions: **1a** (0.1 mmol, 14.28 mmol L^{-1}), Co-F (working area: 1.0 cm^2), a mixed solution of 1.0 M KOH/dioxane (6:1 v/v, 7 mL), −1.1 V vs. Hg/HgO, RT, 6 h.

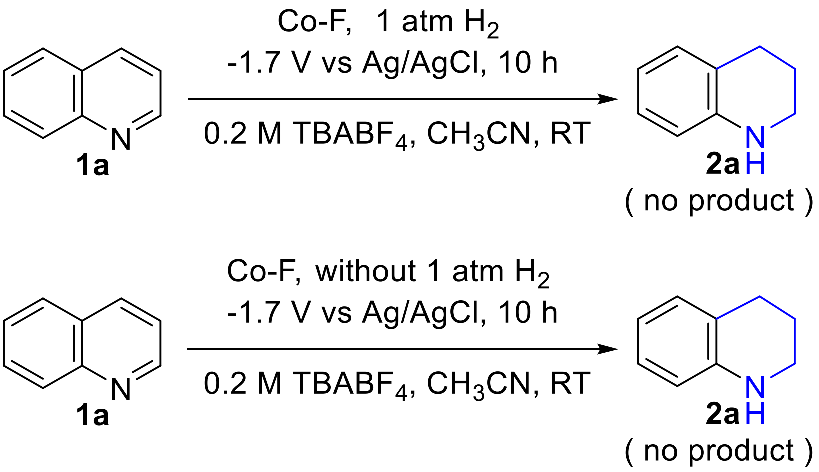

Supplementary Figure 19. Electrocatalytic hydrogenation of 1a in anhydrous CH_3CN in the absence and presence of H_2 over Co-F. Reaction conditions: **1a** (0.1 mmol, 14.28 mmol L^{-1}), Co-F (working area: 1.0 cm^2), with and without H_2, 0.2 M $TBABF_4$ in CH_3CN, −1.7 V vs. Ag/AgCl, RT, 10 h.

A proposed more favorable 1,4/2,3 addition pathway

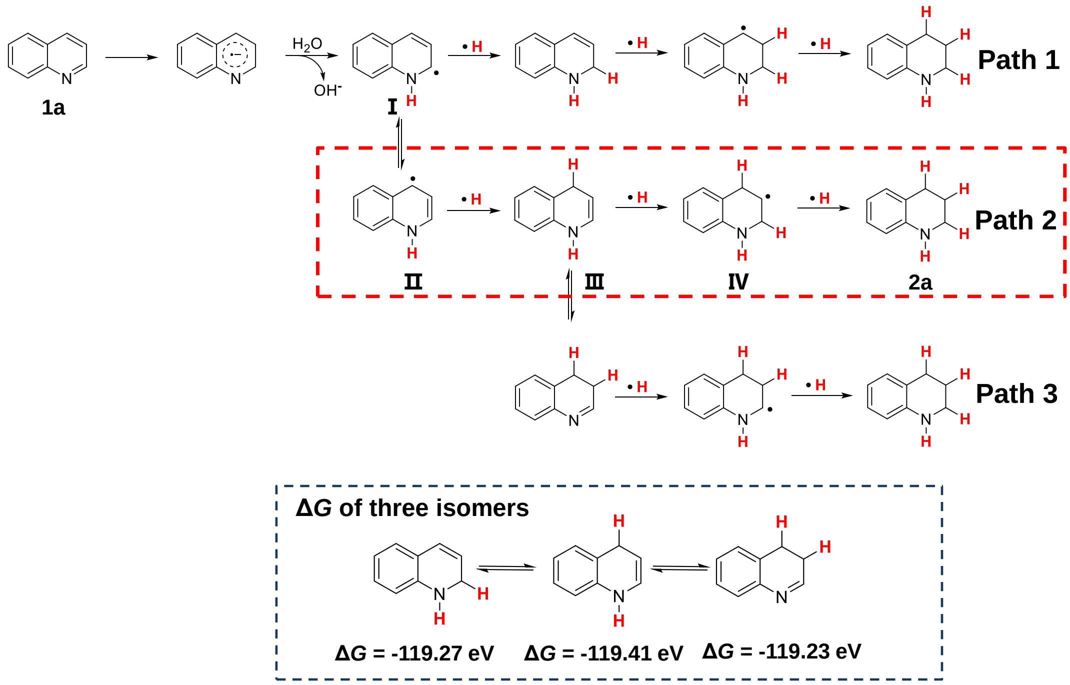

Supplementary Figure 20. Proposed reaction mechanism. Three proposed pathways for electrocatalytic hydrogenation of quinolines with H_2O over the Co-F cathode (**1a** selected as the example).

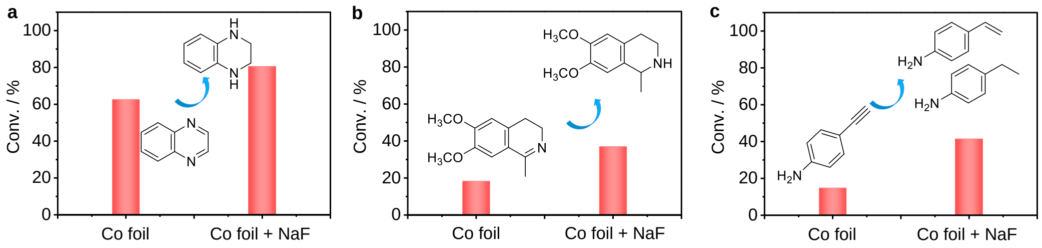

Supplementary Figure 21. Performance of Co foil with and without adding NaF for other electrocatalytic hydrogenation reaction. a Conv. of quinoxaline over Co foil cathode with and without NaF. **b** Conv. of 6,7-dimethoxy-1-methyl-3,4-dihydroisoquinoline over Co foil cathode with and without NaF. **c** Conv. of 4-ethynylaniline over Co foil cathode with and without NaF. Reaction conditions: substrate (0.1 mmol, 14.28 mmol L^{-1}), with and without NaF, Co foil (working area: 1.0 cm^2), a mixed solution of 1.0 M KOH/dioxane (6:1 v/v, 7 mL), RT, **a** −1.2 V vs. Hg/HgO, 2 h; **b** −1.25 V vs. Hg/HgO, 4 h; **c** −1.3 V vs. Hg/HgO, 4 h.

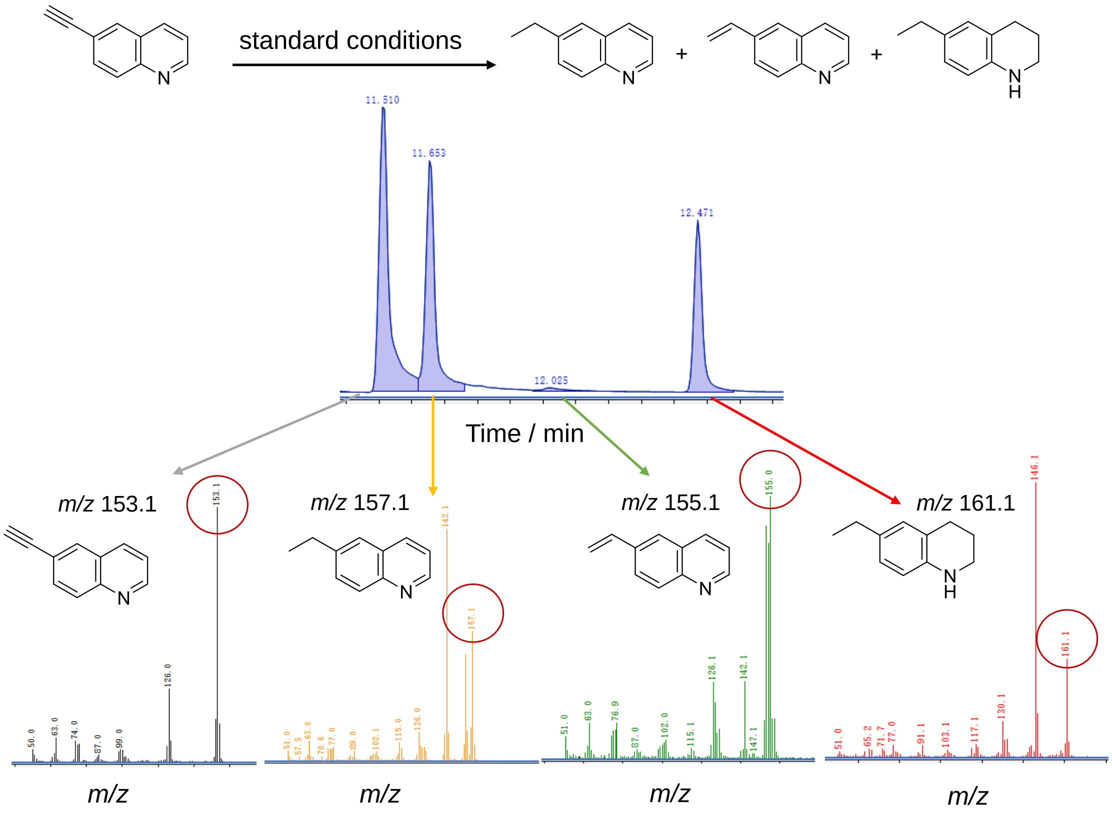

Supplementary Figure 22. A qualitative analysis of possible products by GC-MS for electrocatalytic hydrogenation of 6-ethynylquinoline. Reaction conditions: **6-ethynylquinoline** (0.2 mmol, 28.57 mmol L^{-1}), Co-F (working area: 1.0 cm^2), a mixed solution of 1.0 M KOH/dioxane (5:2 v/v, 7 mL), −1.1 V vs. Hg/HgO, RT, 12 h.

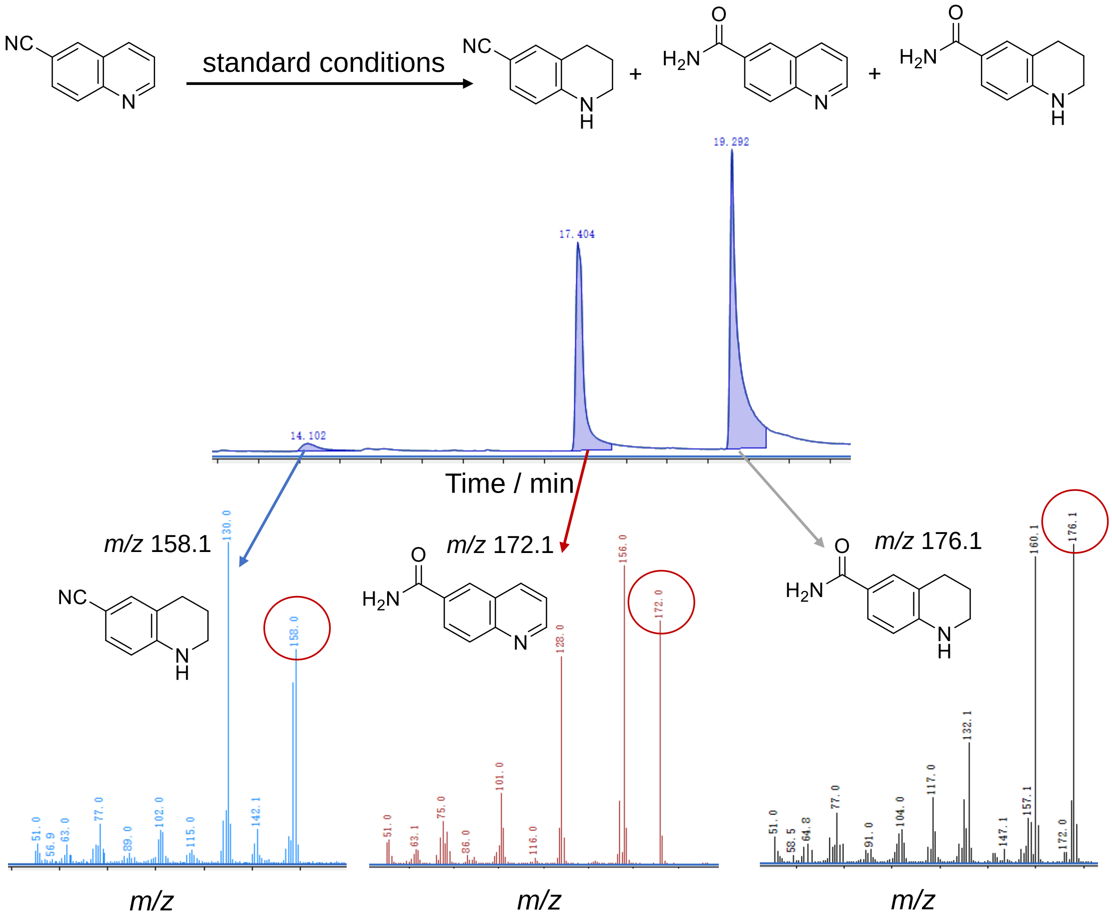

Supplementary Figure 23. A qualitative analysis of possible products by GC-MS for electrocatalytic hydrogenation of quinoline-6-carbonitrile. Reaction conditions: **quinoline-6-carbonitrile** (0.2 mmol, 28.57 mmol L^{-1}), Co-F (working area: 1.0 cm^2), a mixed solution of 1.0 M KOH/dioxane (5:2 v/v, 7 mL), −1.1 V vs. Hg/HgO, RT, 12 h.

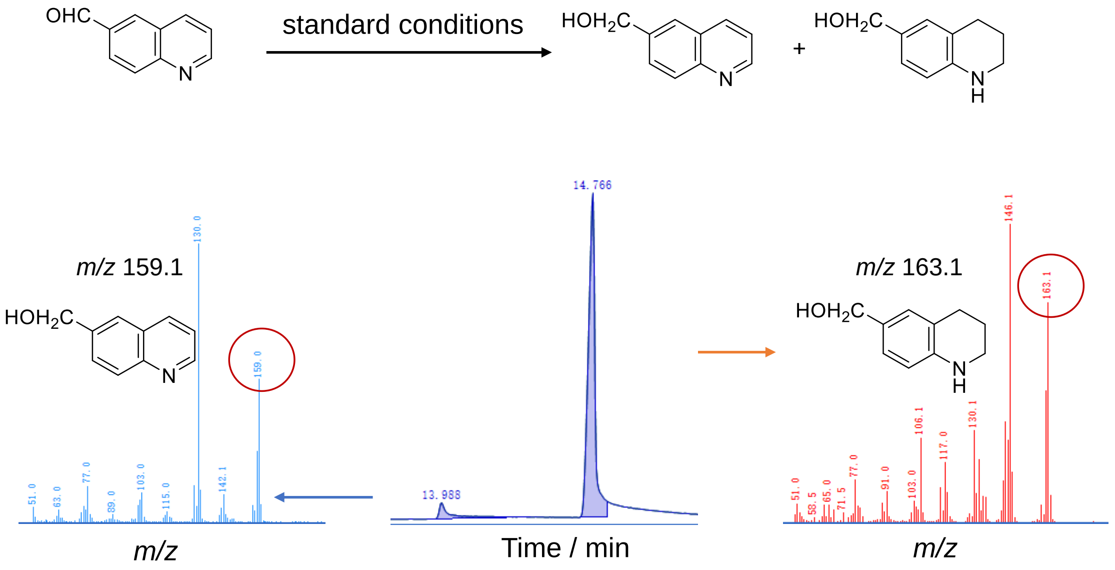

Supplementary Figure 24. **A qualitative analysis of possible products by GC-MS for electrocatalytic hydrogenation of quinoline-6-carbaldehyde.** Reaction conditions: **quinoline-6-carbaldehyde** (0.2 mmol, 28.57 mmol L^{-1}), Co-F (working area: 1.0 cm^2), a mixed solution of 1.0 M KOH/dioxane (6:1 v/v, 7 mL), −1.1 V vs. Hg/HgO, RT, 12 h.

.

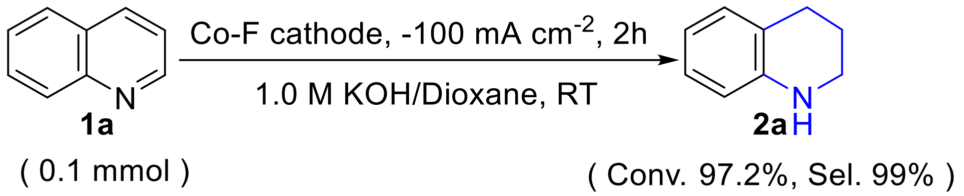

Supplementary Figure 25. Electrocatalytic hydrogenation of 1a with H_2O over Co-F at a constant current density of −100 mA cm^{-2}. Reaction conditions: **1a** (0.1 mmol, 14.28 mmol L^{-1}), Co-F (working area: 1.0 cm^{2}), a mixed solution of 1.0 M KOH/dioxane (6:1 v/v, 7 mL), −100 mA cm^{-2}, RT, 2 h.

Supplementary Table 1. EXAFS fitting parameters of a Co-F catalyst.

**sample**	**path**	**R**	**CN**	**σ^2**	**ΔE_0**	**R-factor**
Co-F	Co-O	2.07(*0.02*)	1.4 (*0.5*)	0.0047(*0.0008*)	1.10(*0.01*)	0.017
	Co-Co	2.49(*0.01*)	6.5 (*0.7*)	0.0027(*0.00098*)	1.10(*0.01*)	

R is the interatomic distance; CN is the coordination number; ΔE_0 is the inner potential shift； σ^2 is the Debye-Waller factor, and R-factor represents the goodness of fit. Roman numbers correspond to fixed values, and uncertainties are given in italic.

Supplementary Table 2. pH values of different electrolytes (6.0 mL) with and without adding dioxane (1.0 mL).

	without dioxane	with dioxane
1.0 M KOH	13.60	14.05
0.5 M K_2CO_3	12.33	12.83
0.5 M K_2HPO_4	9.18	9.55

Supplementary Table 3. Comparisons of the hydrogenation of quinolines to 1,2,3,4-tetrahydroquinolines between representative heterogeneous catalytic methods over non-noble metal catalysts and our method over Co-F.

Catalyst	*n* / mmol	Solvent	Temperature / ℃	H_2 Pressure / bar	Time / h	Conv. / %
Co@C[1]	0.2	i-PrOH	80	20	4	98.6
$Mn(CO)_5Br$[2]	0.5	THF	45	15	18	>99
Co-pyromellitic acid@SiO_2-800[3]	0.5	i-PrOH:H_2O; 2:1	70	10	24	97
CoW@C-0.05[4]	0.53	toluene	100	8	6	>99
Fe-P_{900}-PCC[5]	1	heptane	150	40	12	93
$CoCl_2$/NaBH[6]	0.78	H_2O	130	30	17	>99
CoCu[7]	1	THF	60	40	15	97
Co-SA/AC@ N-CNTs-L[8]	0.5	EtOH	100	20	3	97.4
ISAS-Co/ OPNC[9]	0.5	toluene	120	-	1.5	99
CoO_x@CN[10]	0.5	MeOH	120	30	3	100
Co_3O_4-Co/NGr@ α-Al_2O_3[11]	0.5	toluene	120	20	48	>99
**Co-F (this work)**	**1**	**H_2O/dioxane**	**RT**	**-**	**4**	**98.1**

Supplementary Note 1.

Chemicals and materials. Cobalt nitrate hexahydrate ($Co(NO_3)_2$ $6H_2O$), ammonium fluoride (NH_4F), and urea are purchased from Aladdin Ltd., Shanghai, China. All chemicals are analytical grade and used as received without further purification. Nickel foam (NF) is provided by Canrd. with the purity of 99.99%. Deionized water (DIW) is used in all experiments.

Pretreatment of NF. Firstly, the commercial NF is cut into pieces with the size of 4.0 × 3.0 cm^2. Then, the pieces of NF are ultrasonicated with acetone, water, and 3.0 M HCl aqueous solution for 5 min, respectively. After that, the NF is rinsed with DIW and anhydrous alcohol. Finally, the NF is quickly dried for use under ambient conditions.

Pretreatment of Pt foil, Co foil, Cu foil. The pretreatment procedures of the metallic materials used in this paper are the same as those of NF.

Supplementary Note 2.

Characterizations of Co(OH)F. Scanning electron microscopy (SEM) images (Supplementary Figs. 2a, b) reveal the Co(OH)F with nanowire morphology grow uniformly on NF support. Transmission electron microscopy (TEM) images display a very smooth surface of Co(OH)F and a well-resolved lattice fringe with an interplanar distance of 0.265 nm in the high-resolution TEM (HRTEM) image is indexed to the (201) plane of Co(OH)F (Supplementary Figs. 2c, d). The energy-dispersive X-ray spectroscopy (EDS) elemental mapping verifies the uniform dispersions of Co, O, and F in the sample (Supplementary Fig. 2e). Furthermore, the XPS spectrum is adopted to confirm the valence state of Co and F. In the Co *2p* XPS spectra of Co(OH)F, the deconvoluted peaks located at 782.6 and 797.8 eV are assigned to Co^{2+} $2p_{3/2}$ and Co^{2+} $2p_{1/2}$, while those located at 780.8 and 796.5 eV are attributed to Co^{3+} $2p_{3/2}$ and Co^{3+} $2p_{1/2}$ (Fig. 1d). And, F 1*s* spectrum at 684.05 eV is ascribed to F^{-} (Fig. 1e). Moreover, all the diffraction peaks in the X-ray diffraction (XRD) pattern are indexed to Co(OH)F (JCPDS NO. 50-0827) (Supplementary Fig. 5a).

Supplementary Note 3.

GC measurements. Gas chromatography (GC) is use for the sample quantification analysis of the hydrogenation reactions. After the reaction is finished, dodecane was added in the reaction system as an internal standard. Then the cathodic products are extracted with ethyl acetate (EA). The extracted solutions are dried with anhydrous

sodium sulfate and then are concentrated under reduced pressure on the rotary evaporator to remove the solvent. After re-dissolving with EA, it is analyzed by GC. The spectra are obtained with a gas chromatograph (Agilent 7890) equipped with HP-5 capillary column (30 m×250 μm), thermal conductivity (TCD) and a flame ionization detector (FID). The injection temperature was set at 300 ℃. Nitrogen was used as the carrier gas at 1.5 mL min^{-1}. The initial column temperature is 50 ℃ and then increases to 250 ℃ at 10 ℃ min^{-1}, finally keeping at 250 ℃ for 5 min. A 1.0 μL of sample is taken out for detection by GC.

Supplementary Note 4.

Product quantifications. To be more specific, "Conv." is calculated via dividing the amount (millimoles) of consumed substrate by originally added millimoles, and " Sel." is calculated via dividing the obtained millimoles of product by the consumed millimoles of substrate. In addition, the isolated yields of the products in the Table 1 and Fig. 5a are are provided, which are calculated via dividing the obtained millimoles of the hydrogenated products or deuterated products by the the initial added substrates to the reactor.

Supplementary Note 5.

EPR experiments of electrocatalytic hydrogenation of 1a. The hydrogen radicals and carbon radicals are trapped by the addition of 5,5-dimethyl-1-pyrroline-*N*-oxide (DMPO) to the cathodic cell containing 7.0 mL 0.5 M Na_2SO_4 electrolyte. A Co-F NW working electrode and a Hg/HgO reference electrode are put into the cathode chamber, and a graphite rod counter electrode is inserted into the anode chamber. Chronoamperometry is carried out at a given constant potential of −1.1 V vs. Hg/HgO for 10 min with 0.1 mmol **1a**. Then, 0.1 mM DMPO is added and stirred for 1 min. After that, the solution is quickly taken out for the EPR test.

Supplementary Note 6.

***t*-BuOH trapping H radical experiments.** To ensure the reliability of the comparison experiment, the Co-F cathode is cut into two halves with the same area. Then, they are used as the working electrodes for electrocatalytic hydrogenation of **1a** under the standard reaction conditions at −1.1 V vs. Hg/HgO with and without *t*-BuOH, respectively, for 6 h. The content of **1a** is detected every 30 minutes. 2 batches of 100 μL of *t*-BuOH are added to the cathodic compartment at 0 h, 2.5 h, respectively.

Supplementary Note 7.

The peak at 7.26 ppm in ^{1}H NMR spectra is assigned to the hydrogen signal of $CDCl_3$. Three peaks around 77 ppm in ^{13}C NMR spectra are assigned to the carbon signal of $CDCl_3$. **2r-2t**, **5a** are not isolated for NMR analysis, and their GC-MS data are provided. Since the N–D bonds in the product are relatively active, H/D exchange is likely to occur during post-processing. Therefore, the deuterated ratios at the C–D positions of the products are reported. Furthermore, some carbon atoms are missed in the ^{13}C spectra of the deuterated products due to the presence of D, which are consistent with the reported literature.[12-15]

Supplementary Note 8.

General procedure for gram-scale electrocatalytic hydrogenation of 1a and 1b by using H_2O as the hydrogen source. Electrocatalytic measurements were carried out in a divided three-electrode system consisting of a working electrode (working area: 3.0×3.0 cm^2), a carbon rod counter-electrode, and a Hg/HgO (1.0 M KOH) reference electrode. The cathodic cell (40 mL) and anodic cell (40 mL) containing 30 mL of 1.0 M KOH solution, respectively, are separated by the membrane. **1a** or **1b** and 6 mL dioxane are added into the cathodic cell and stirred to form a homogeneous solution. Then chronoamperometry is carried out at −1.2 V vs. Hg/HgO until the reaction is finished (20 h for **1a** and 32 h for **1b**).

Supplementary Note 9.

The procedure for paired reactions at both Co-F cathode and NiSe anode. In a divided Co-F||NiSe two-electrode system, 0.2 mmol quinoline (**1a**) and 1,6-hexanediamine (**4a**) are added into the cathodic and anodic cell, respectively. Then, the reaction is carried out at −2.2 V for 12 h. About 97.2 % and 97.5 % conversions are respectively achieved. The selectivity of tetrahydroquinoline **2a** and adiponitrile **5a** both reach to 99%.

^{1}H NMR, ^{13}C NMR spectra, and GC-MS data

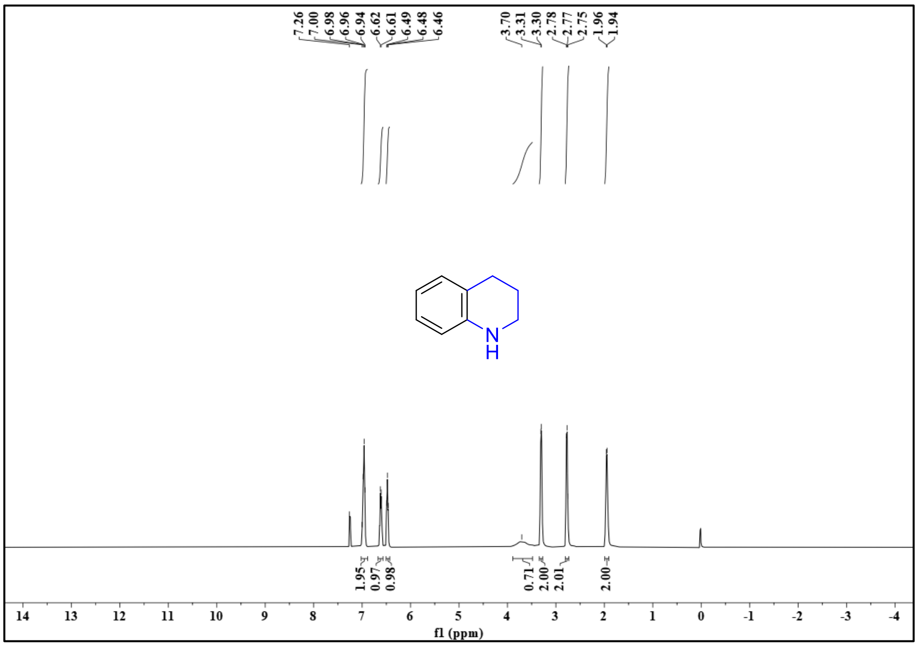

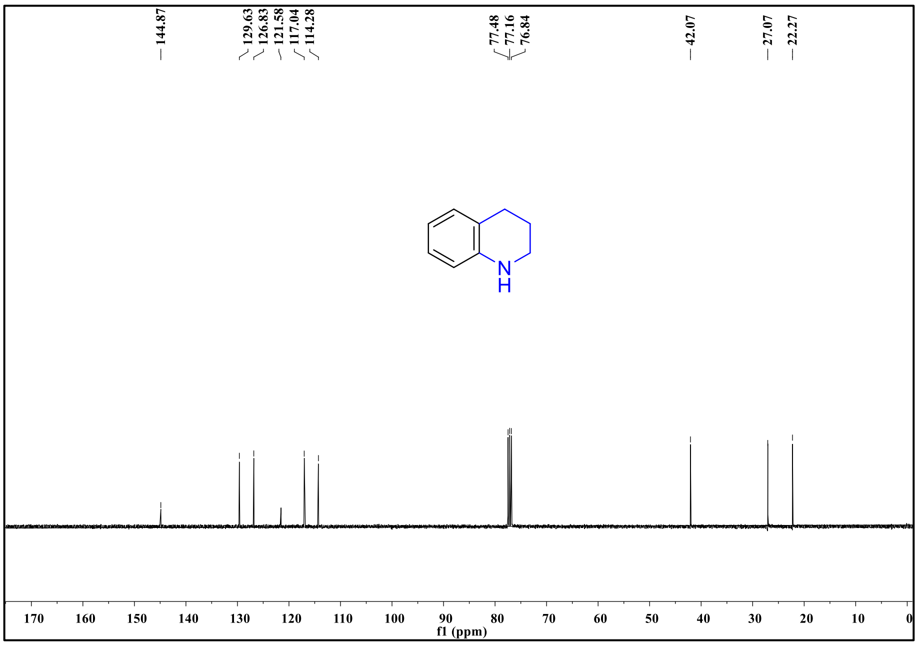

Supplementary Figure 26. **^{1}H NMR** (400 MHz, $CDCl_3$) δ [ppm] 6.97 (dd, J = 16.0, 8.0 Hz, 2H), 6.61 (d, J = 7.0 Hz, 1H), 6.48 (t, J = 6.5 Hz, 1H), 3.70 (s, 1H), 3.35 – 3.25 (m, 2H), 2.77 (t, J = 6.5 Hz, 2H), 2.01 – 1.89 (m, 2H); **^{13}C NMR** (101 MHz, $CDCl_3$) δ [ppm] 144.87, 129.63, 126.83, 121.58, 117.04, 114.28, 42.07, 27.07, 22.27; **GC–MS** (EI) $[M-H]^-$ 132.1, theoretical value for $C_9H_{11}N$ m/z 133.1.

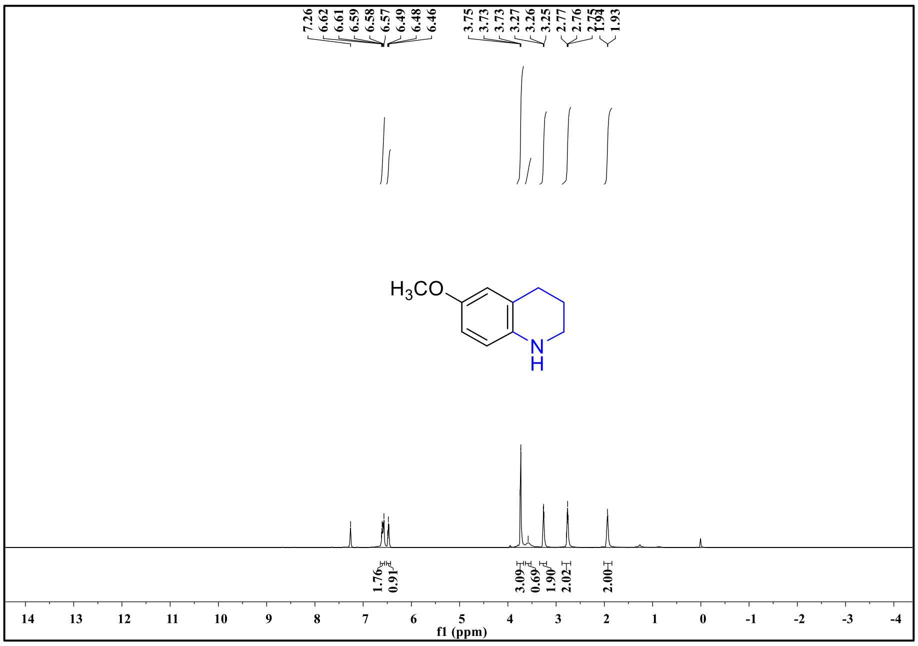

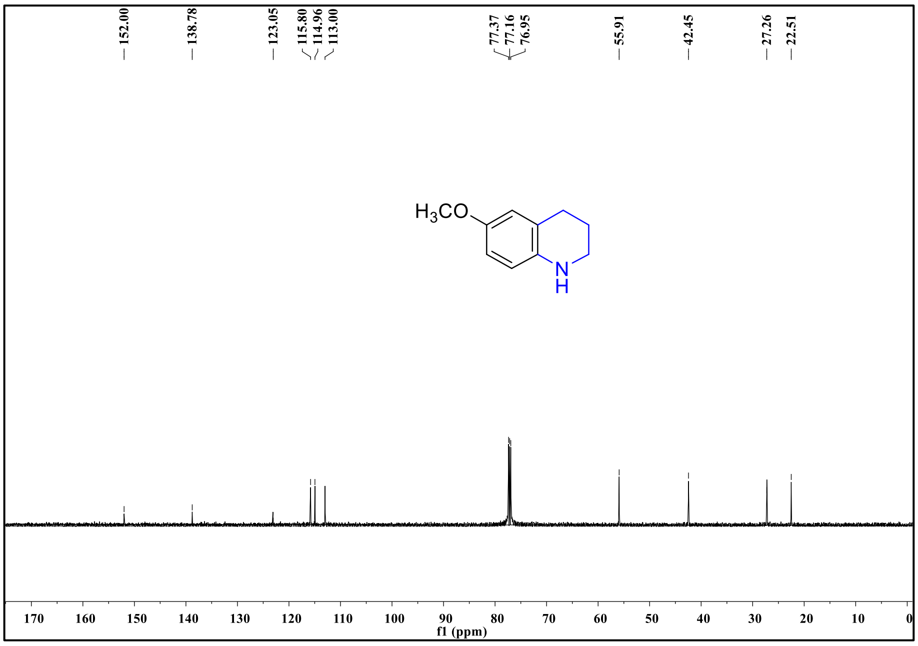

Supplementary Figure 27. ^{1}**H NMR** (400 MHz, $CDCl_3$) δ [ppm] 6.62 – 6.57 (m, 2H), 6.51 – 6.45 (d, J = 8.2 Hz, 1H), 3.75 (s, 3H), 3.58 (s, 1H), 3.26 (m, 2H), 2.77 (t, J = 6.5 Hz, 2H), 1.95 – 1.92 (m, 2H); ^{13}**C NMR** (101 MHz, $CDCl_3$) δ [ppm] 152.00, 138.78, 123.05, 115.80, 114.96, 113.00, 55.91, 42.45, 27.26, 22.51; **GC–MS** (EI) [M] 163.1, theoretical value for $C_{10}H_{13}NO$ m/z 163.1.

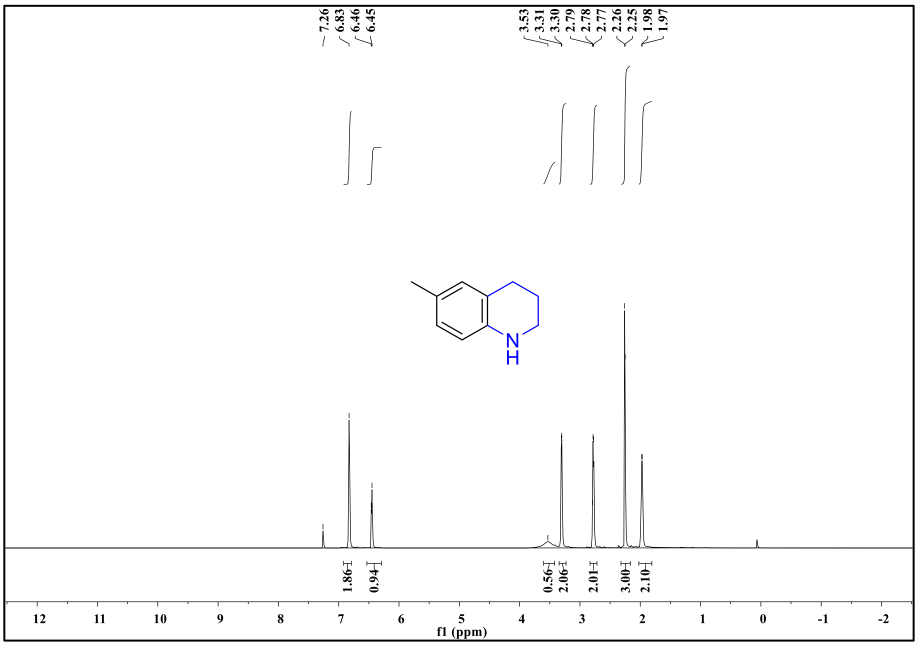

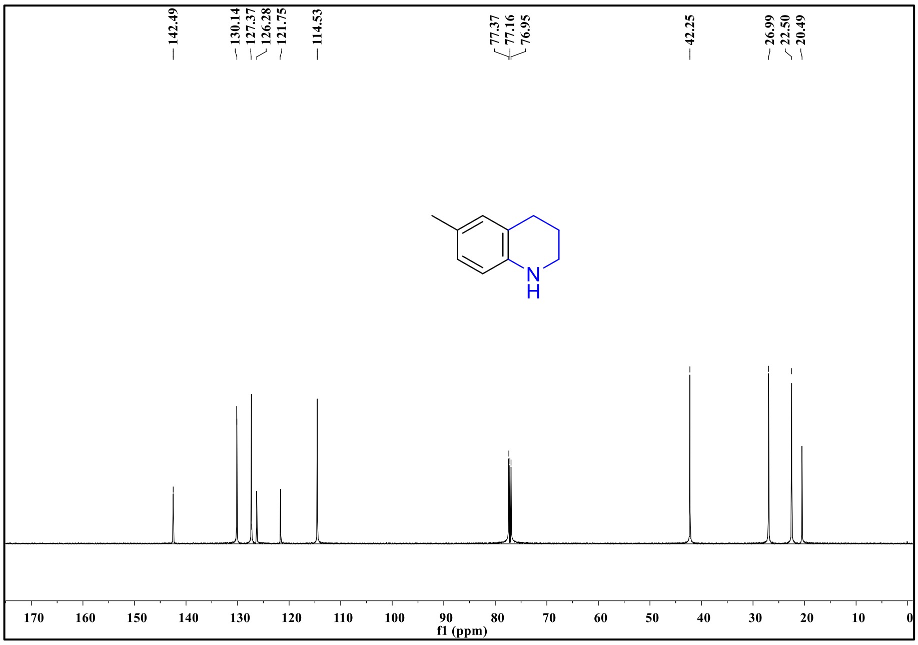

Supplementary Figure 28. ^{1}**H NMR** (400 MHz, $CDCl_3$) δ [ppm] 6.83 – 6.78 (m, 2H), 6.45 – 6.39 (m, 1H), 3.53 (s, 1H), 3.31 – 3.28 (m, 2H), 2.77 (t, J = 6.6 Hz, 2H), 2.25 (s, 3H), 1.97 (m, 2H); ^{13}**C NMR** (101 MHz, $CDCl_3$) δ [ppm] 142.49, 130.14, 127.37, 126.28, 121.75, 114.53, 42.25, 26.99, 22.50, 20.49; **GC–MS** (EI) $[M\text{-}H]^-$ 146.1, theoretical value for $C_{10}H_{13}N$ m/z 147.1.

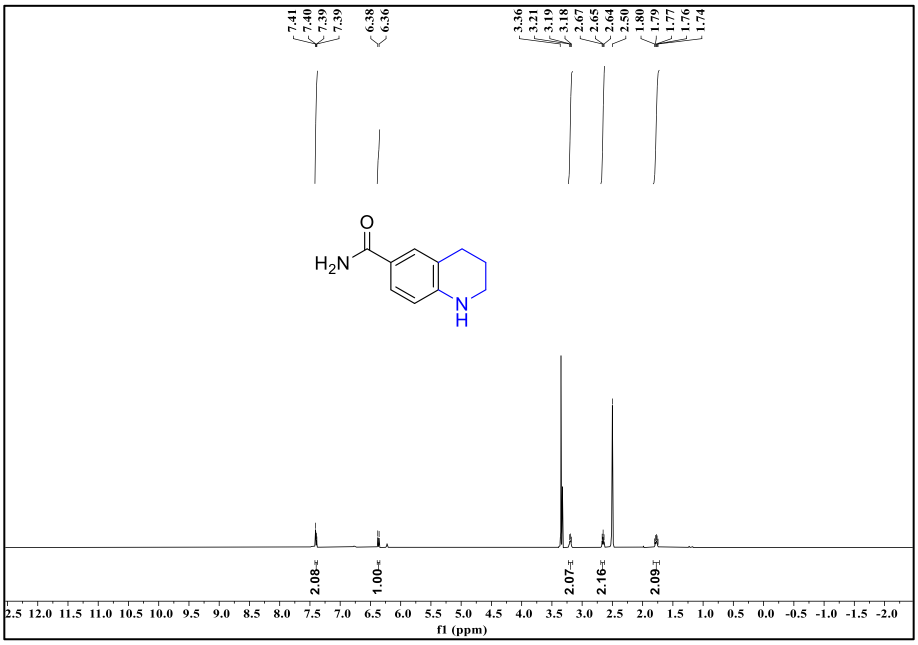

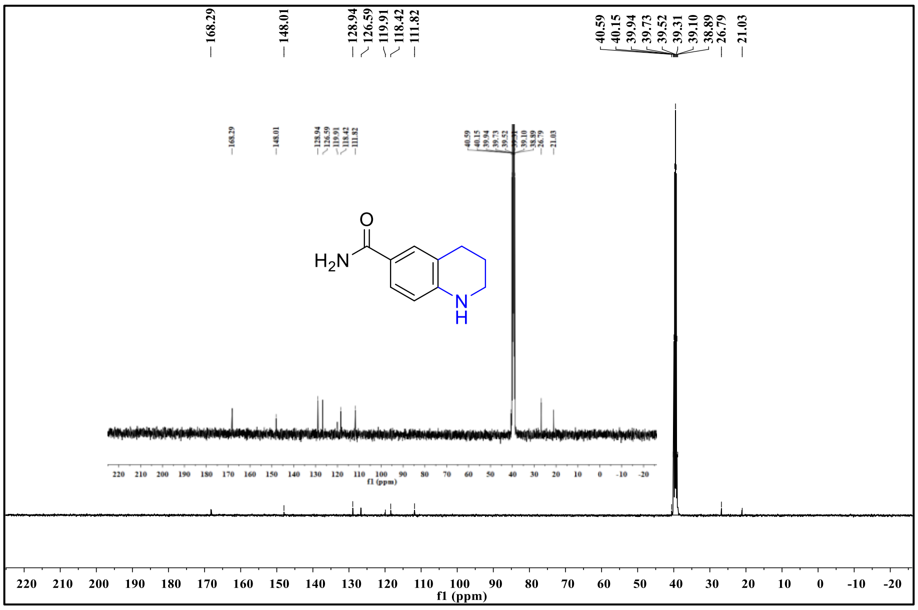

Supplementary Figure 29. **^{1}H NMR** (400 MHz, $(CD_3)_2SO$) δ [ppm] 7.40 (m, 2H), 6.37 (d, J = 9.0 Hz, 1H), 3.19 (t, J = 5.6 Hz, 2H), 2.65 (t, J = 6.3 Hz, 2H), 1.77 (dt, J = 11.1 Hz, 6.2 Hz, 2H); **^{13}C NMR** (101 MHz, $(CD_3)_2SO$) δ [ppm] 168.29, 148.01, 128.94, 126.59, 119.91, 118.42, 111.82, 40.59, 26.79, 21.03; **GC–MS** (EI) [M] 176.1, theoretical value for $C_{10}H_{12}N_2O$ *m/z* 176.1.

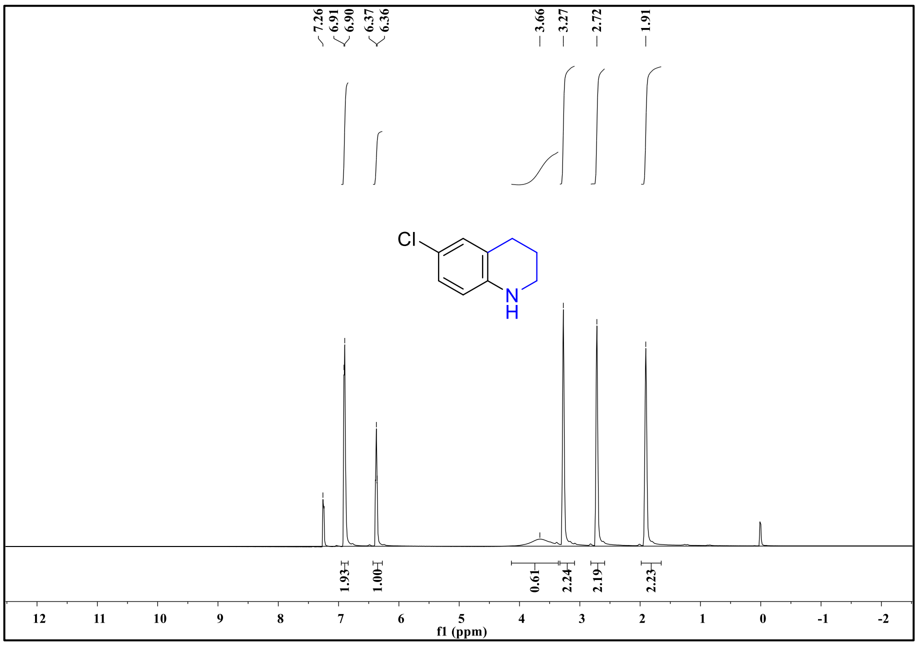

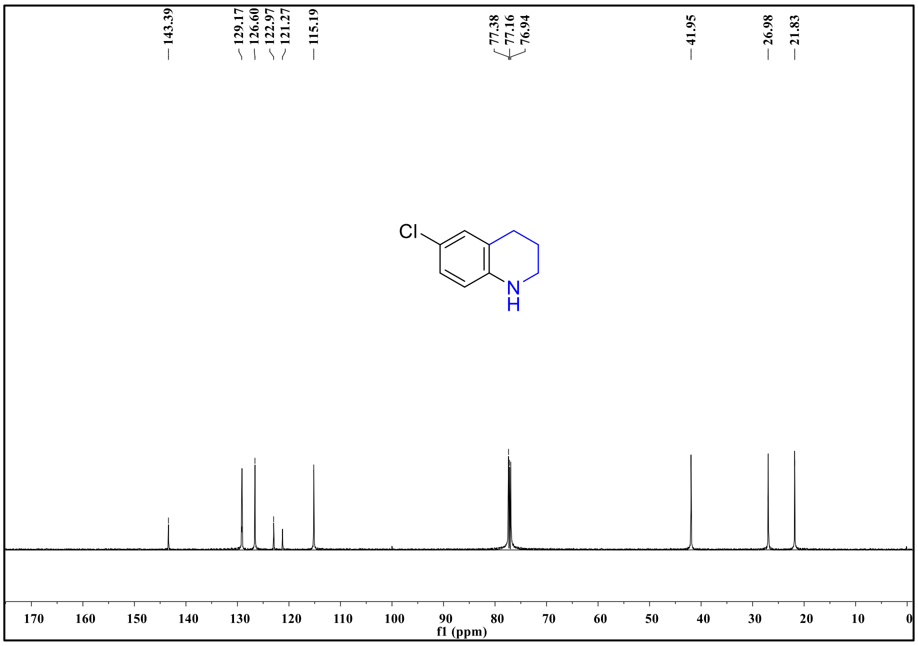

Supplementary Figure 30. ^{1}**H NMR** (400 MHz, $CDCl_3$) δ [ppm] 6.90 (m, 2H), 6.37 – 6.35 (m, 1H), 3.66 (s, 1H), 3.27 (m, 2H), 2.73 – 2.71 (t, 2H), 1.91 – 1.89 (m, 2H); ^{13}**C NMR** (101 MHz, $CDCl_3$) δ [ppm] 143.39, 129.17, 126.60, 122.97, 121.27, 115.19, 41.95, 26.98, 21.83; **GC–MS** (EI) [M] 167.1, theoretical value for $C_9H_{10}ClN$ *m/z* 167.1.

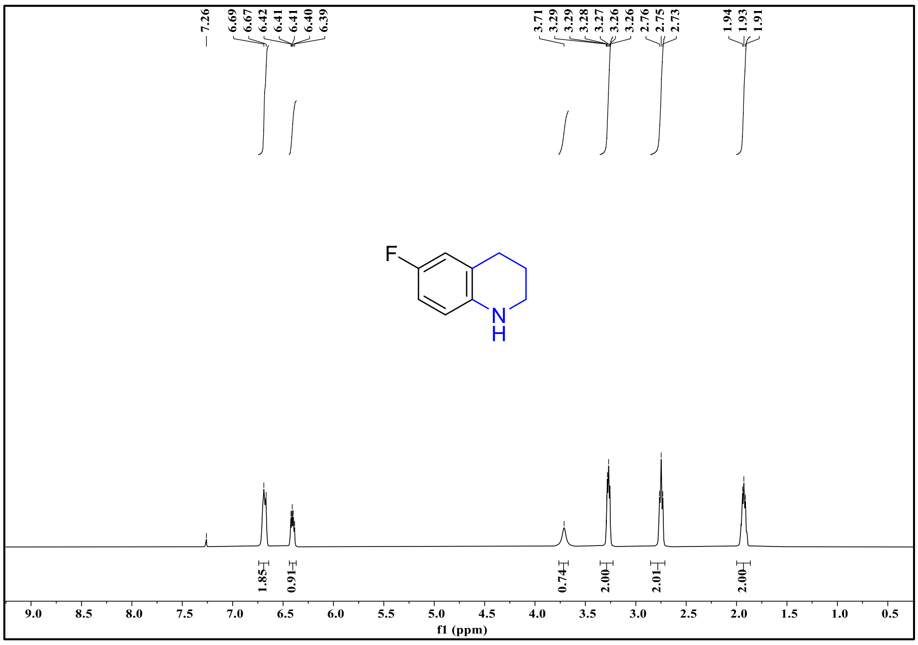
7.26
6.69
6.67
6.42
6.41
6.41
6.40
6.39
3.71
3.29
3.29
3.28
3.27
3.26
3.26
2.76
2.75
2.73
1.94
1.93
1.91
F
N
H
1.85
0.91
0.74
2.00
2.01
2.00
9.0 8.5 8.0 7.5 7.0 6.5 6.0 5.5 5.0 4.5 4.0 3.5 3.0 2.5 2.0 1.5 1.0 0.5
f1 (ppm)

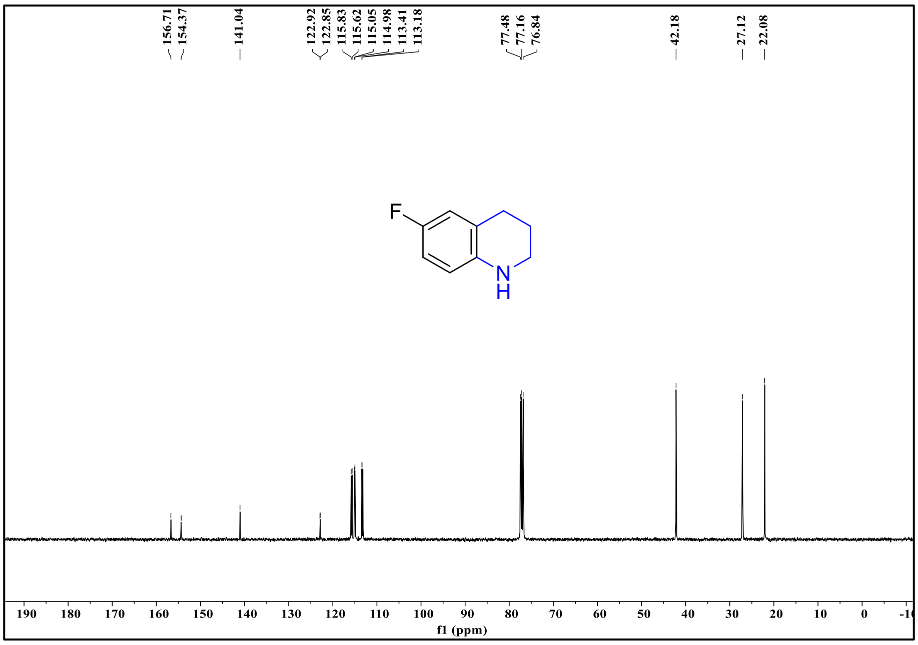
156.71
154.37
141.04
122.92
122.85
115.83
115.62
115.05
114.98
113.41
113.18
77.48
77.16
76.84
42.18
27.12
22.08
F
N
H
190 180 170 160 150 140 130 120 110 100 90 80 70 60 50 40 30 20 10 0
f1 (ppm)

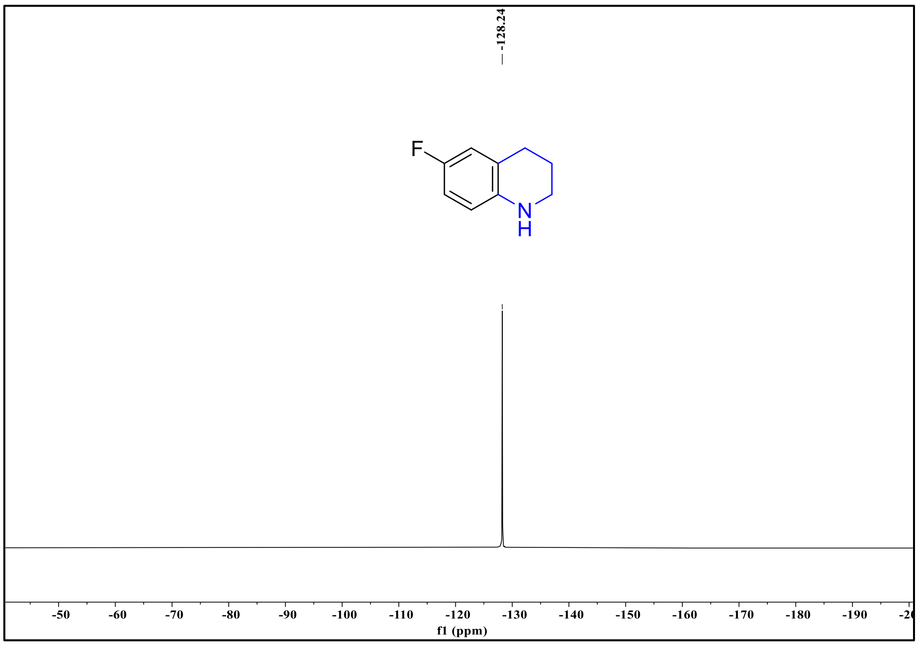

Supplementary Figure 31. **^{1}H NMR** (400 MHz, $CDCl_3$) δ [ppm] 6.68 (d, J = 9.3 Hz, 2H), 6.42 – 6.37 (m, 1H), 3.71 (s, 1H), 3.29 – 3.23 (m, 2H), 2.75 (t, J = 6.4 Hz, 2H), 2.00 – 1.86 (m, 2H). **^{13}C NMR** (101 MHz, $CDCl_3$) δ [ppm] 155.54 (d, J = 236.34 Hz), 141.04 (s), 122.885 (d, J = 7.07 Hz), 115.725 (d, J = 21.21 Hz), 115.015 (d, J = 7.07 Hz), 113.295 (d, J = 23.23 Hz), 42.18, 27.12, 22.08; **^{19}F NMR** (376 MHz, $CDCl_3$) δ [ppm] −128.24 (s, 1F); **GC–MS** (EI) [M] 151.1, theoretical value for $C_9H_{10}FN$ *m/z* 151.1.

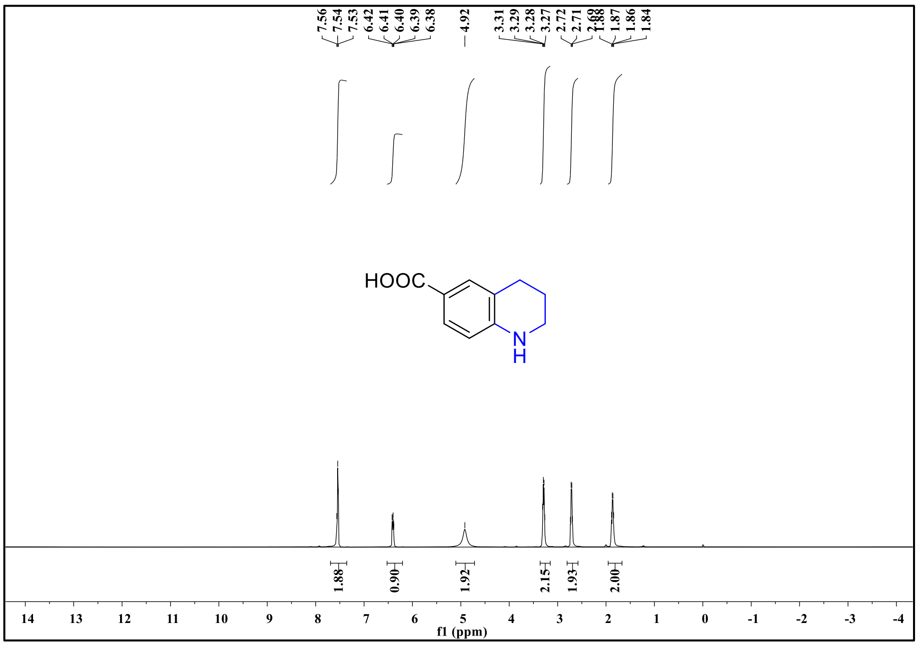

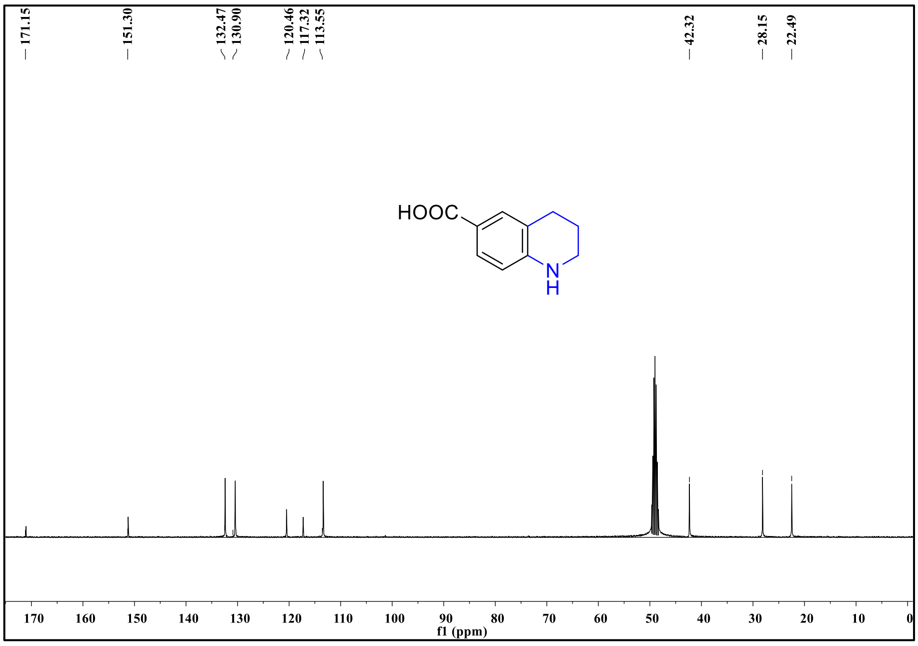

Supplementary Figure 32. **^{1}H NMR** (400 MHz, CD_3OD) δ [ppm] 7.58 – 7.52 (m, 2H), 6.45 – 6.36 (d, J = 9.1, 1H), 4.92 (s, 2H), 3.32 – 3.25 (m, 2H), 2.71 (t, J = 6.3 Hz, 2H), 1.89 – 1.82 (m, 2H); **^{13}C NMR** (101 MHz, CD_3OD) δ [ppm] 171.15, 151.30, 132.47, 130.90, 120.46, 117.32, 113.55, 42.32, 28.15, 22.49. GC–MS (EI) [M] 177.1, theoretical value for $C_{10}H_{11}NO_2$ m/z 177.1.

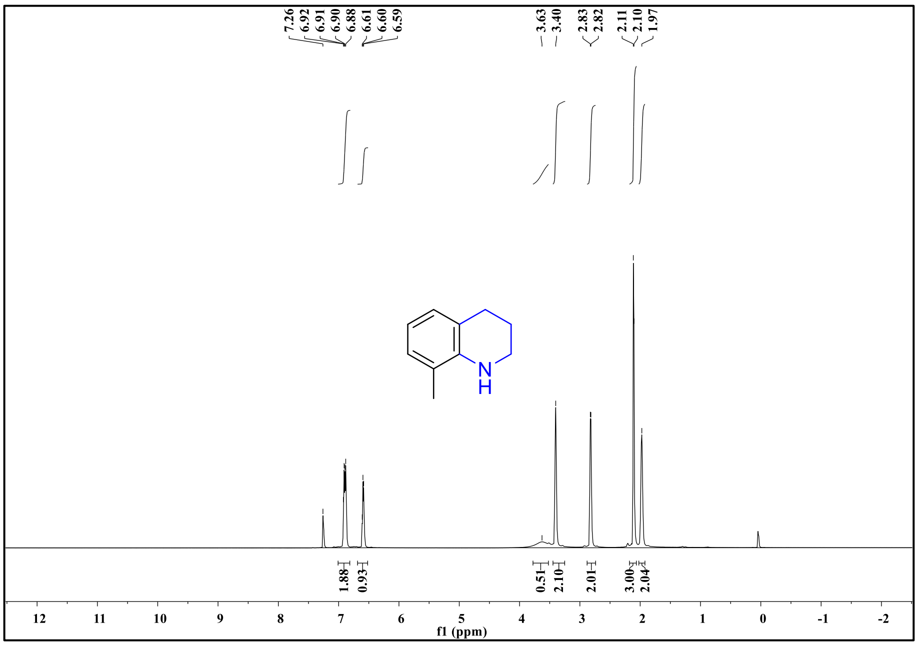

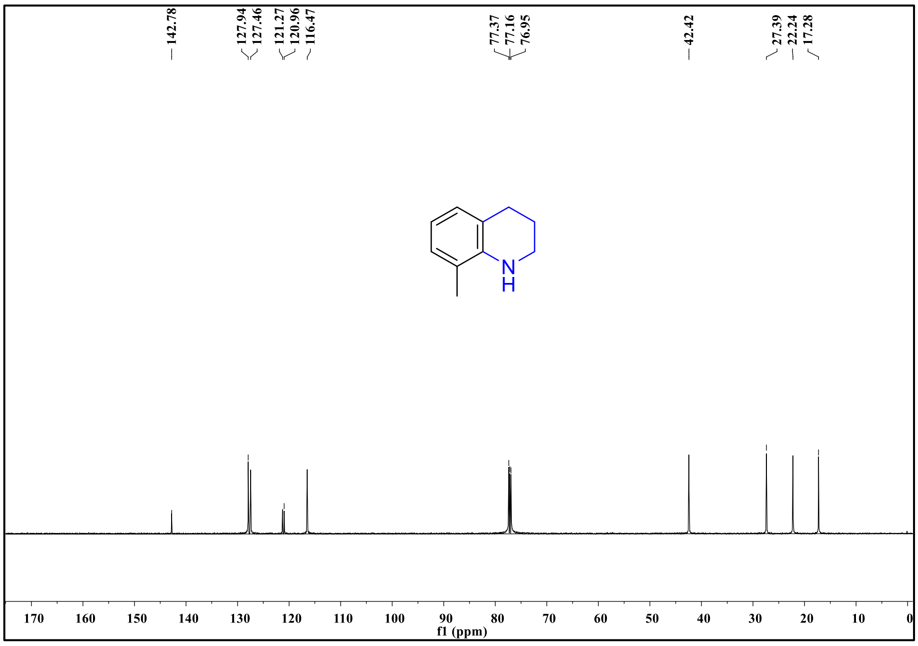

Supplementary Figure 33. **^{1}H NMR** (400 MHz, $CDCl_3$) δ [ppm] 6.92 (dd, J = 17.2, 7.5 Hz, 2H), 6.60 (t, J = 7.5 Hz, 1H), 3.63 (s, 1H), 3.42 – 3.39 (m, 2H), 2.83 (t, J = 6.4, 2H), 2.11 (s, 3H), 1.97 (m, 2H); **^{13}C NMR** (101 MHz, $CDCl_3$) δ [ppm] 142.78, 127.94, 127.46, 121.27, 120.96, 116.47, 42.42, 27.39, 22.24, 17.28; **GC–MS** (EI) $[M-H]^-$ 146.1, theoretical value for $C_{10}H_{13}N$ m/z 147.1.

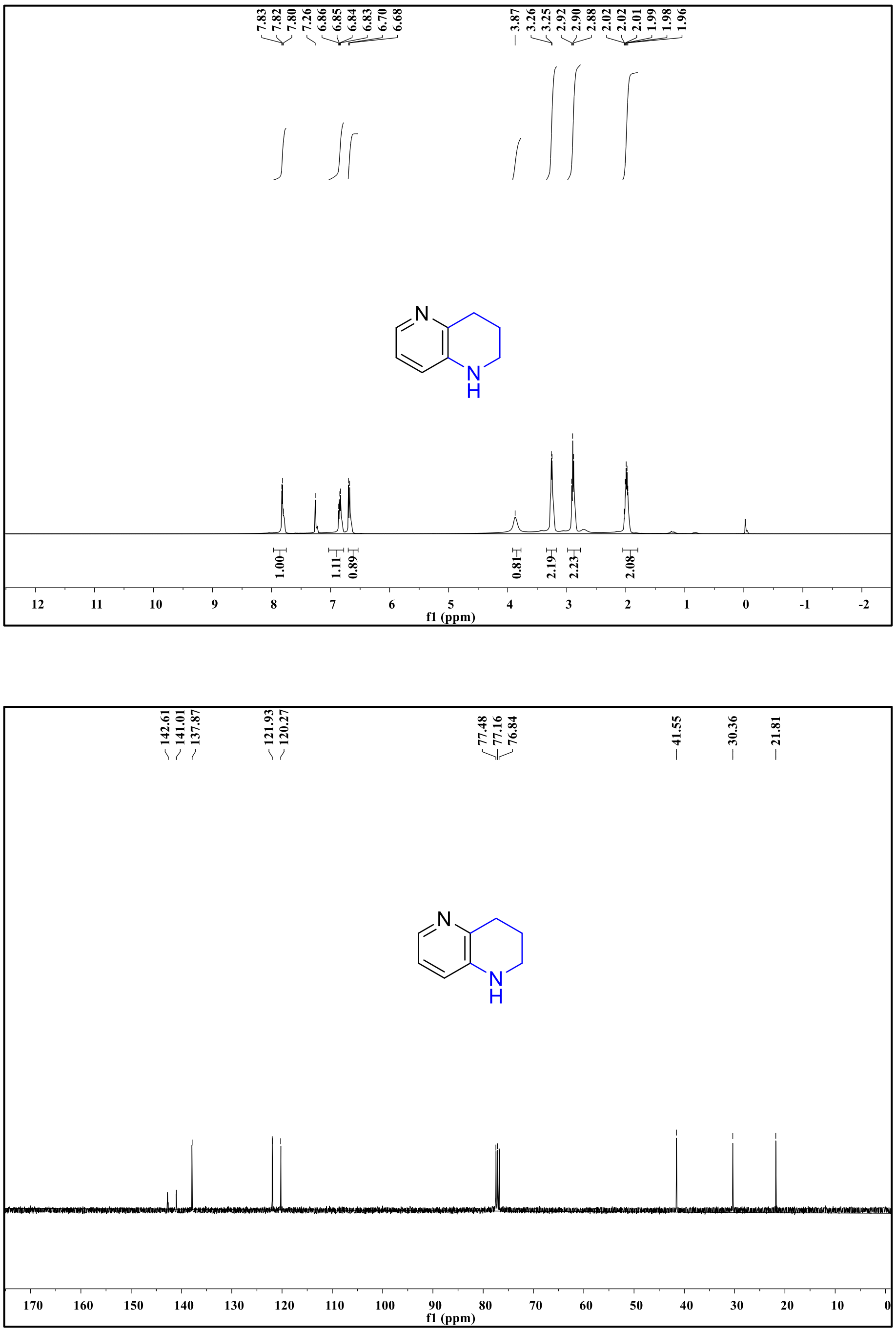

Supplementary Figure 34. ^{1}H NMR (400 MHz, $CDCl_3$) δ [ppm] 7.83 (d, J = 4.8 Hz, 1H), 6.86 – 6.83 (dd, J = 8.3, 4.9 Hz, 1H), 6.71 – 6.68 (d, J = 8.1 Hz,1H), 3.87 (s, 1H), 3.26 (m, 2H), 2.92 – 2.88 (t, J = 6 Hz, 2H), 2.02 – 1.96 (m, 2H); **^{13}C NMR** (101 MHz, $CDCl_3$) δ [ppm] 142.61, 141.01, 137.87, 121.93, 120.27, 41.55, 30.36, 21.81; **GC–MS** (EI) $[M\text{-}H]^-$ 133.1, theoretical value for $C_8H_{10}N_2$ *m/z* 134.1.

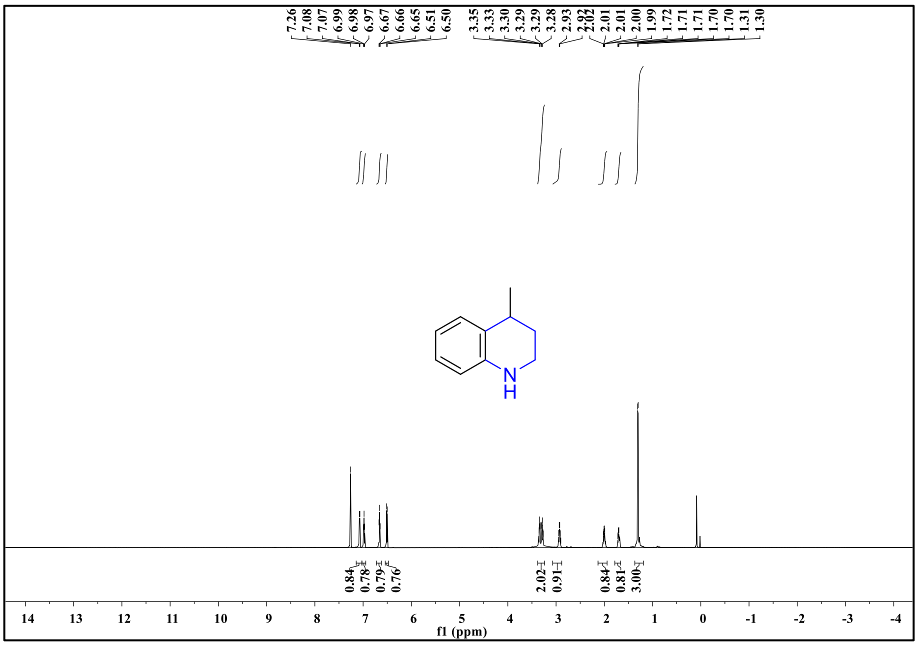

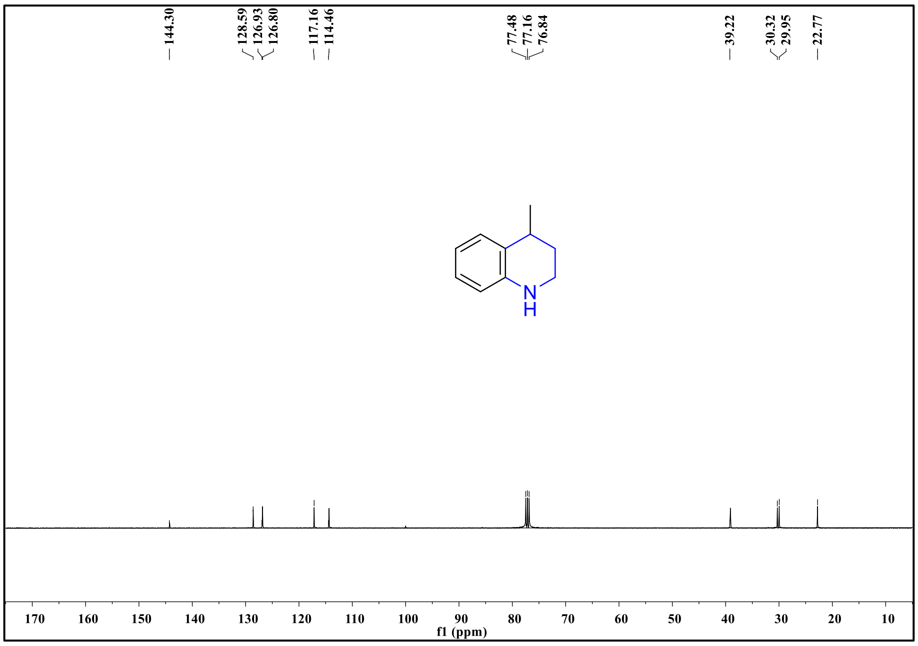

Supplementary Figure 35. **^{1}H NMR** (400 MHz, $CDCl_3$) δ [ppm] 7.07 (d, J = 7.5 Hz, 1H), 6.98 (td, J = 7.8 Hz, 1.6 Hz, 1H), 6.66 (td, J = 7.8 Hz, 1.5 Hz, 1H), 6.51 (d, J = 8.3 Hz, 1H), 3.36 – 3.27 (m, 2H), 2.92 (m, 1H), 2.13 – 1.99 (m, 1H), 1.78 – 1.69 (m, 1H), 1.30 (d, J = 9 Hz, 3H); **^{13}C NMR** (101 MHz, $CDCl_3$) δ [ppm] 144.30, 128.59, 126.93, 126.80, 117.16, 114.46, 39.22, 30.32, 29.95, 22.77; **GC–MS** (EI) [M] 147.1, theoretical value for $C_{10}H_{13}N$ *m/z* 147.1.

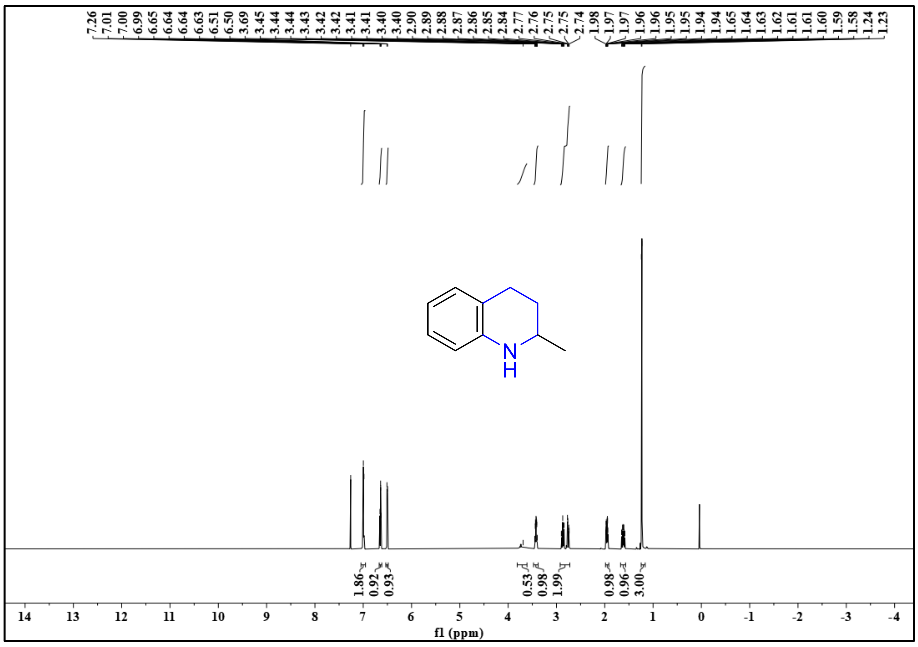

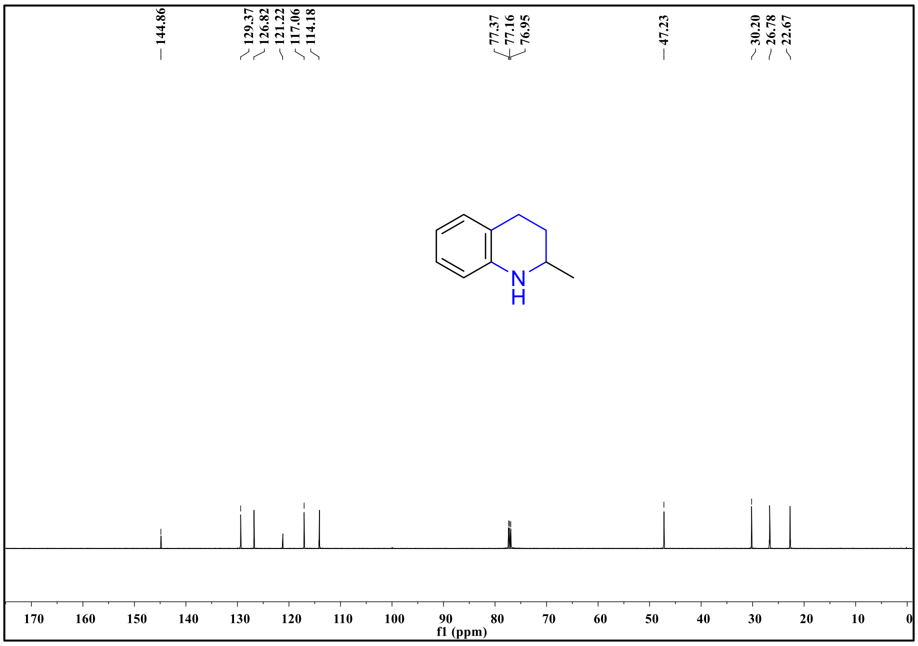

Supplementary Figure 36. ^{1}**H NMR** (400 MHz,$CDCl_3$) δ [ppm] 7.00 (t, J = 4.0 Hz, 2H), 6.64 (t, J = 4.0 Hz, 1H), 6.50 (d, J = 4.0 Hz, 1H), 3.72 – 3.44 (s, 1H), 3.45 – 3.40 (m, 1H), 2.90 – 2.74 (m, 2H), 1.98 – 1.94 (m, 1H), 1.65 – 1.58 (m, 1H), 1.24 (d, J = 4 Hz, 3H); ^{13}**C NMR** (101 MHz, $CDCl_3$) δ [ppm] 144.86, 129.37, 126.82, 121.22, 117.06, 114.18, 47.23, 30.20, 26.78, 22.67; **GC–MS** (EI) [M] 147.1, theoretical value for $C_{10}H_{13}N$ m/z 147.1.

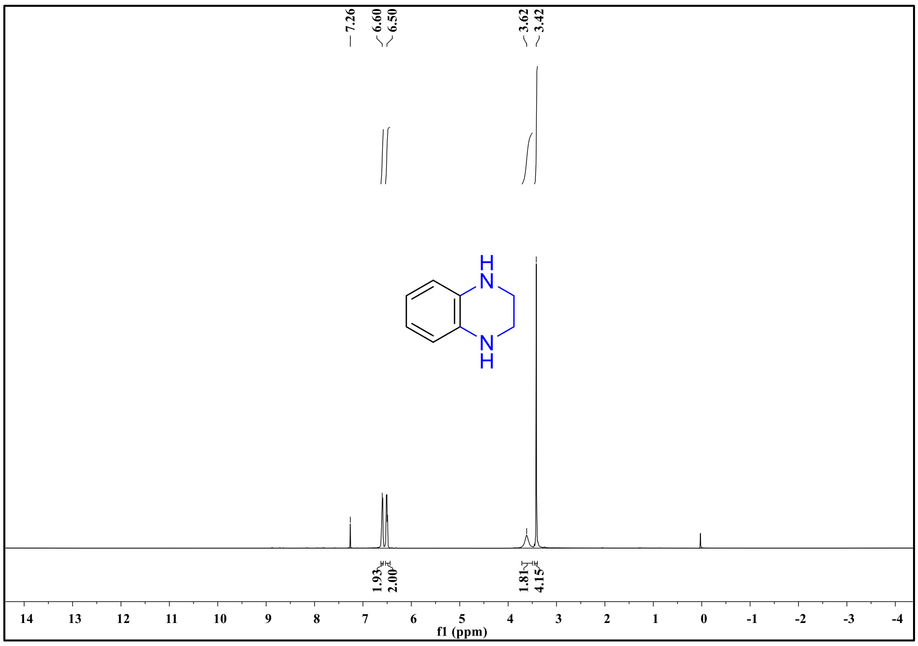

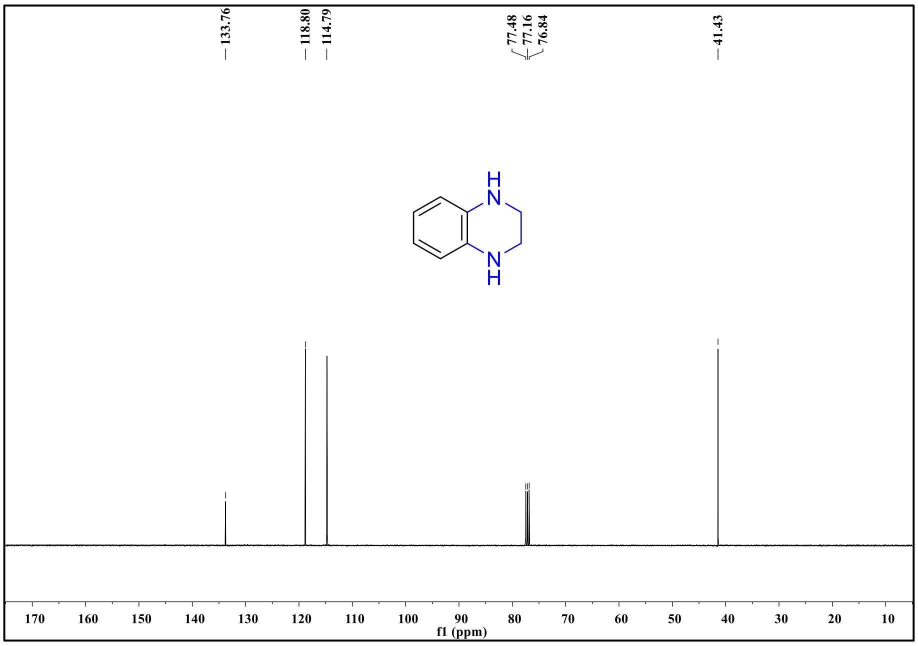

Supplementary Figure 37. ^{1}**H NMR** (400 MHz, $CDCl_3$) δ [ppm] 6.60 (s, 2H), 6.50 (s, 2H), 3.62 (s, 2H), 3.42 (s, 4H); ^{13}**C NMR** (101 MHz, $CDCl_3$) δ [ppm] 133.76, 118.80, 114.79, 41.43; **GC–MS** (EI) $[M\text{-}H]^-$ 133.1, theoretical value for $C_8H_{10}N_2$ *m/z* 134.1.

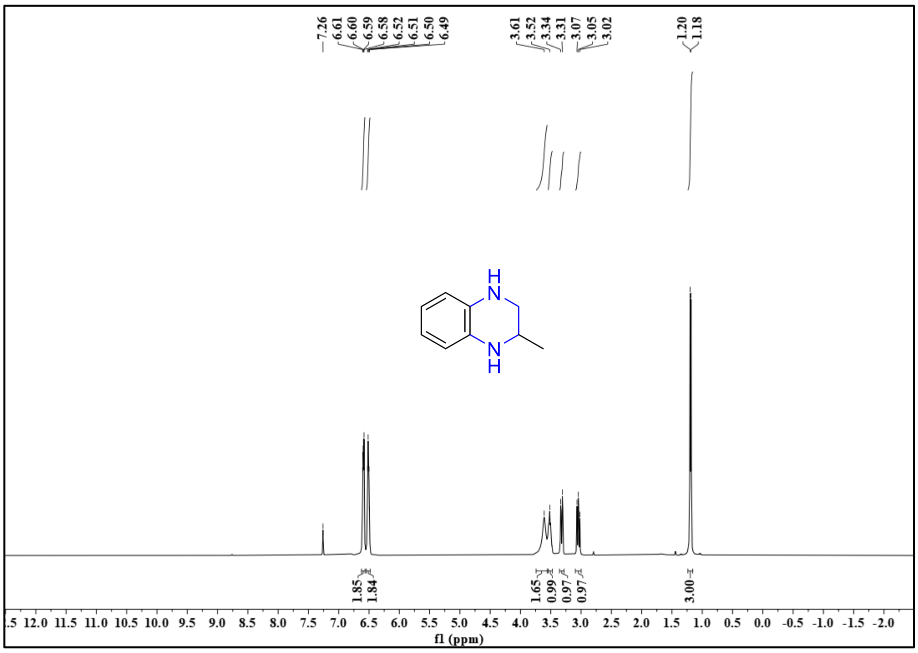

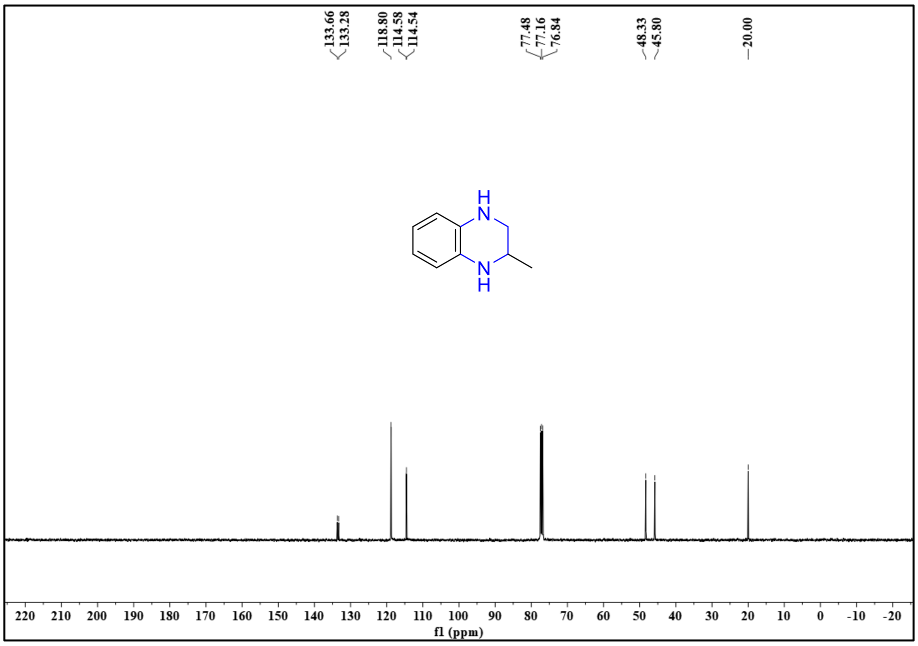

Supplementary Figure 38. **^{1}H NMR** (400 MHz, $CDCl_3$) δ [ppm] 6.59 (dd, J = 5.8 Hz, 3.4 Hz, 2H), 6.55 – 6.48 (m, 2H), 3.6 (s, 2H), 3.55 – 3.48 (m, 1H), 3.34 (d, *J* = 12.0 Hz, 1H), 3.05 (t, *J* = 8.0 Hz, 1H), 1.20 (d, *J* = 8.0 Hz, 3H); **^{13}C NMR** (101 MHz, $CDCl_3$) *δ* [ppm] 133.66, 133.28, 118.80 (2C), 114.58, 114.54, 48.33, 45.80, 20.00; **GC–MS** (EI) [M] 148.1, theoretical value for $C_9H_{12}N_2$ *m/z* 148.1.

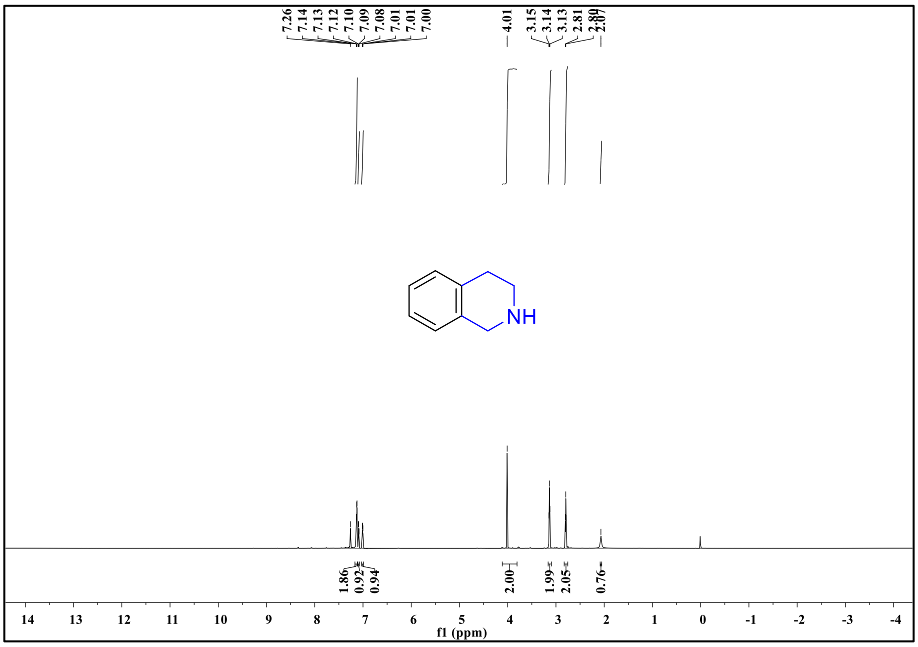

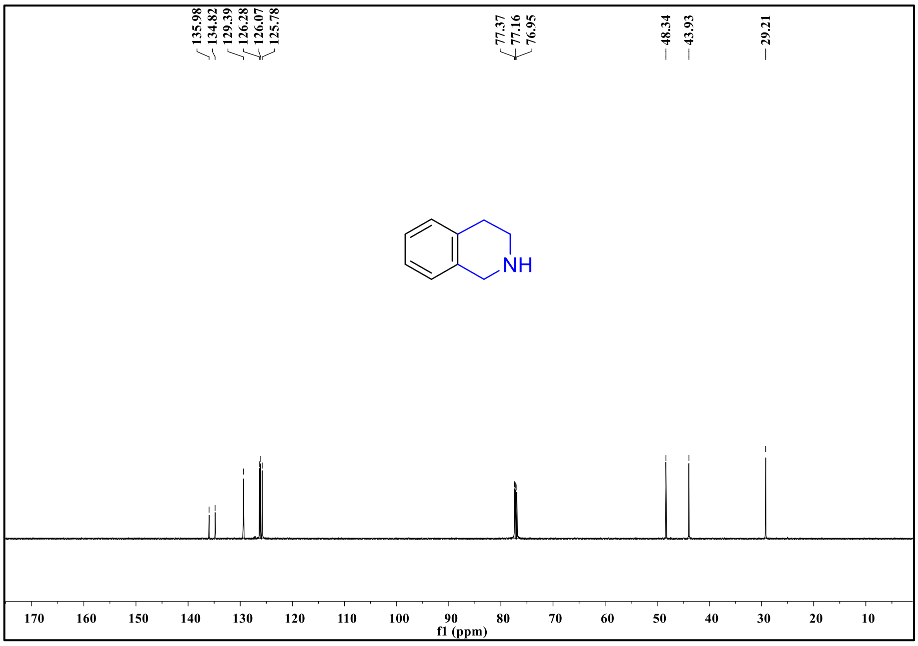

Supplementary Figure 39. **^{1}H NMR** (400 MHz, $CDCl_3$) δ [ppm] 7.14 – 7.08 (m, 2H), 7.09 (dd, J = 8.0, 4.0 Hz, 1H), 7.03 – 6.99 (m, 1H), 4.01 (s, 2H), 3.14 (t, J = 4.0 Hz, 2H), 2.80 (t, J = 4.0 Hz, 2H), 2.07 (s, 1H); **^{13}C NMR** (101 MHz, $CDCl_3$) δ [ppm] 135.98, 134.82, 129.39, 126.28, 126.07, 125.78, 48.34, 43.93, 29.21; **GC–MS** (EI) $[M-H]^-$ 132.1, theoretical value for $C_9H_{11}N$ m/z 133.1.

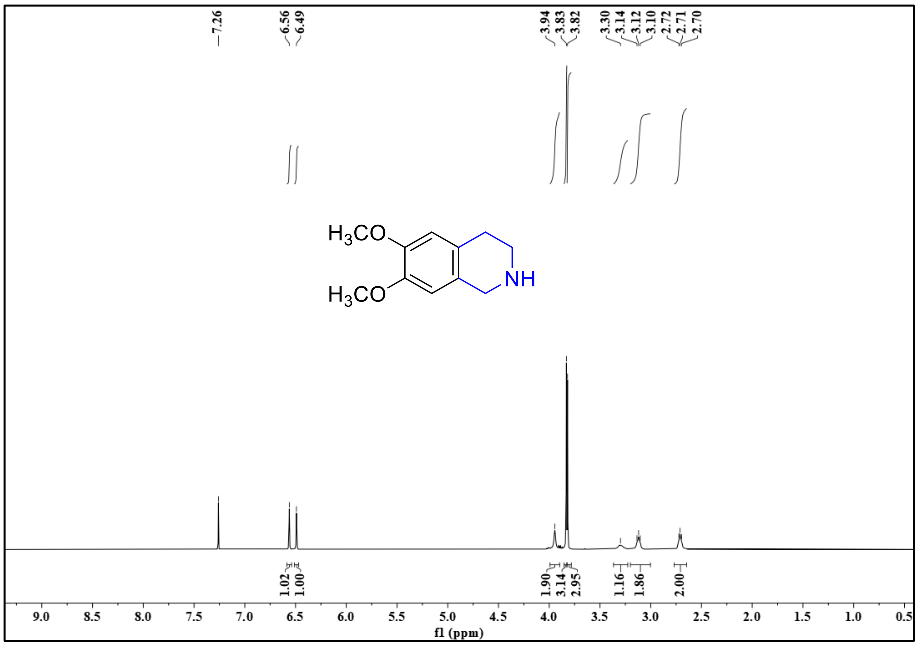

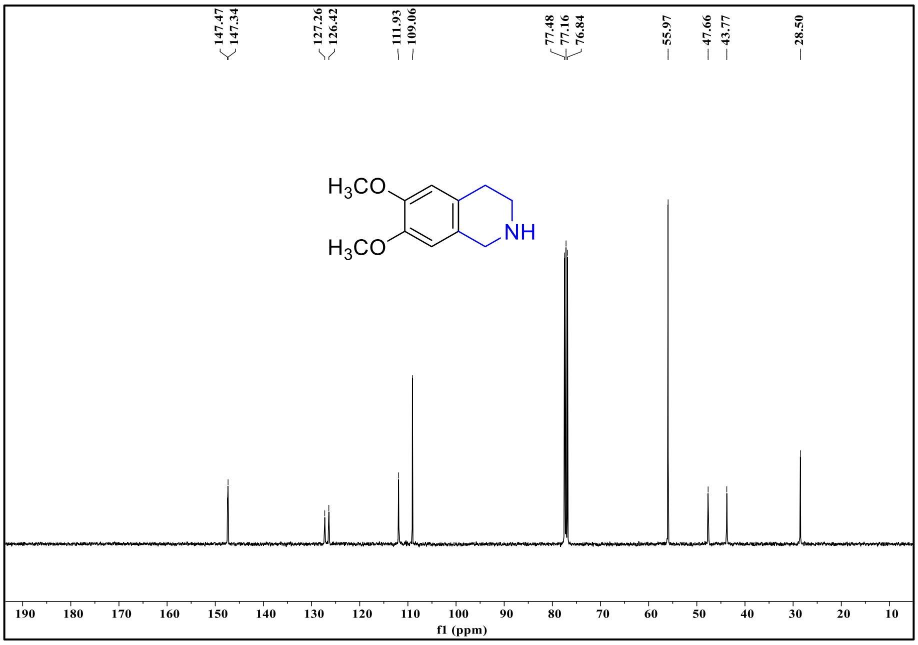

Supplementary Figure 40. ^{1}**H NMR** (400 MHz, $CDCl_3$) δ [ppm] 6.56 (s, 1H), 6.49 (s, 1H), 3.94 (s, 2H), 3.83 (s, 3H), 3.82 (s, 3H), 3.30 (s, 1H), 3.12 (t, J = 8.0 Hz, 2H), 2.71 (t, J = 4.0 Hz, 2H); ^{13}**C NMR** (101 MHz, $CDCl_3$) δ [ppm] 147.47, 147.34, 127.26, 126.42, 111.93, 109.06, 55.97 (2C), 47.66, 43.77, 28.50; **GC–MS** (EI) $[M\text{-}H]^-$ 192.1, theoretical value for $C_{11}H_{15}NO_2$ *m/z* 193.1.

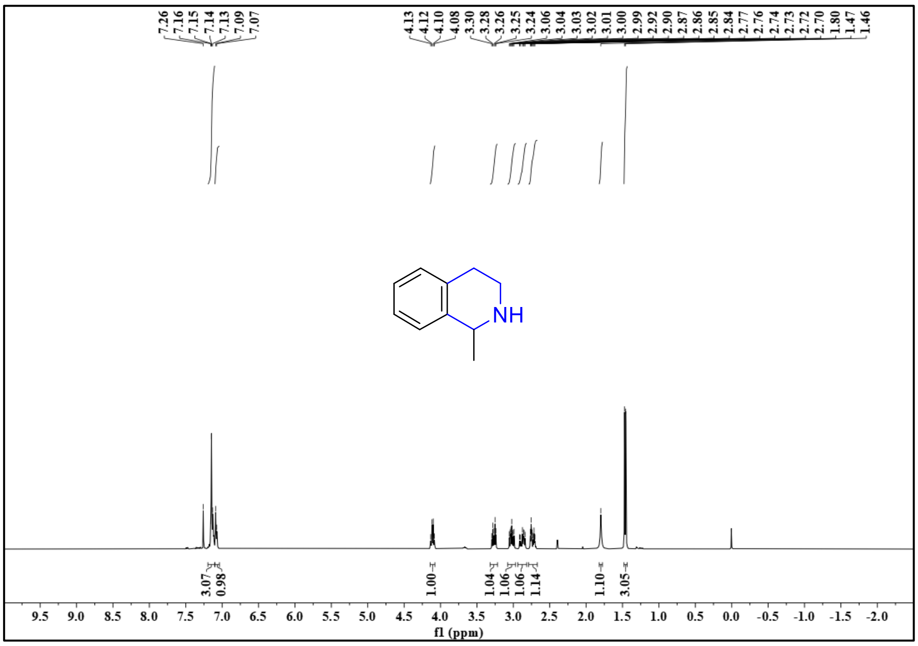

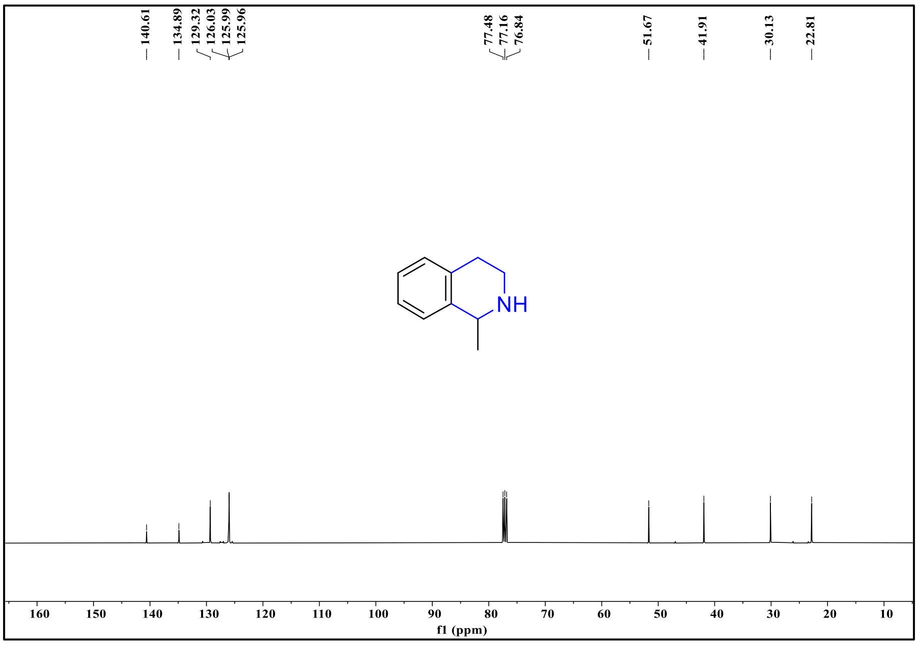

Supplementary Figure 41. ^{1}H NMR (400 MHz, $CDCl_3$) δ [ppm] 7.16 – 7.13 (m, 3H), 7.08 (d, J = 8.0 Hz, 1H), 4.11 (q, J = 4.0 Hz, 1H), 3.30 – 3.24 (m, 1H), 3.06 – 2.98 (m, 1H), 2.94 – 2.83 (m, 1H), 2.74 (dt, J = 16.0 Hz, 4.0 Hz, 1H), 1.80 (s, 1H), 1.47 (d, J = 4.0 Hz, 3H); ^{13}**C NMR** (101 MHz, $CDCl_3$) δ [ppm] 140.61, 134.89, 129.32, 126.03, 125.99, 125.96, 51.67, 41.91, 30.13, 22.81; **GC–MS** (EI) $[M-H]^{-}$ 146.1, theoretical value for $C_{10}H_{13}N$ m/z 147.1.

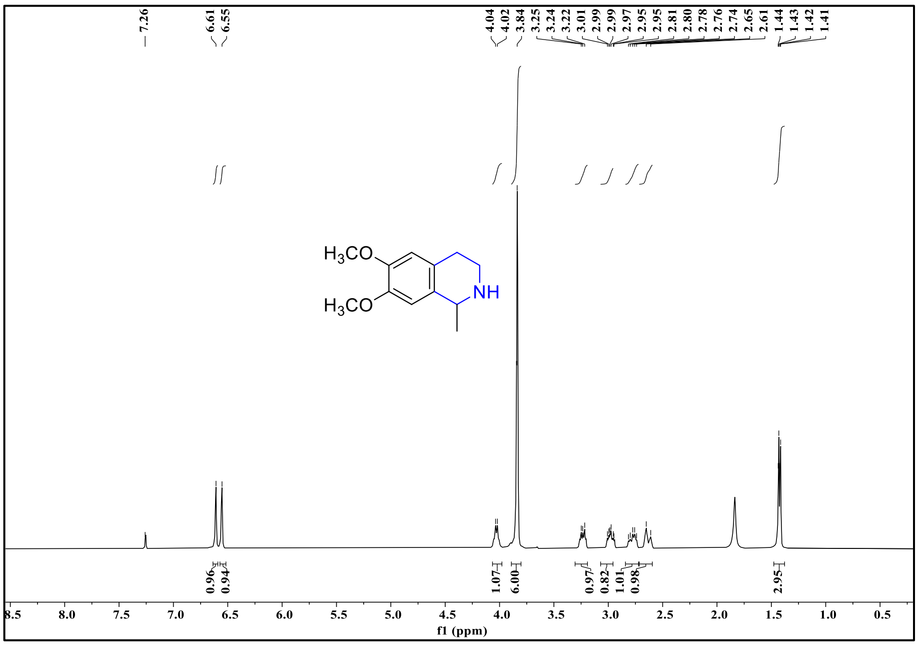

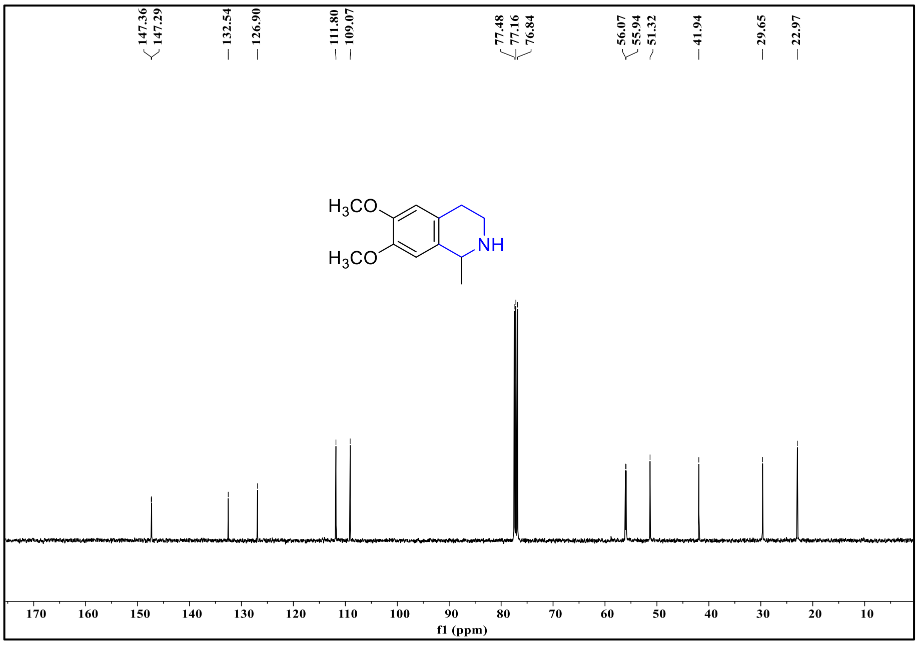

Supplementary Figure 42. ^{1}**H NMR** (400 MHz, $CDCl_3$) δ [ppm] 6.61 (s, 1H), 6.55 (s, 1H), 4.03 (q, J = 6.7 Hz, 1H), 3.84 (s, 6H), 3.30 – 3.20 (m, 1H), 3.04 – 2.94 (m, 1H), 2.78 (dt, J = 14.1, 6.8 Hz, 1H), 2.63 (d, J = 16.4 Hz, 1H), 1.43 (dd, J = 6.6, 2.4 Hz, 3H); ^{13}**C NMR** (101 MHz, $CDCl_3$) δ [ppm] 147.36, 147.29, 132.54, 126.90, 111.80, 109.07, 56.07, 55.94, 51.32, 41.94, 29.65, 22.97; **GC–MS** (EI) $[M\text{-}H]^-$ 206.1, theoretical value for $C_{12}H_{17}NO_2$ *m/z* 207.1.

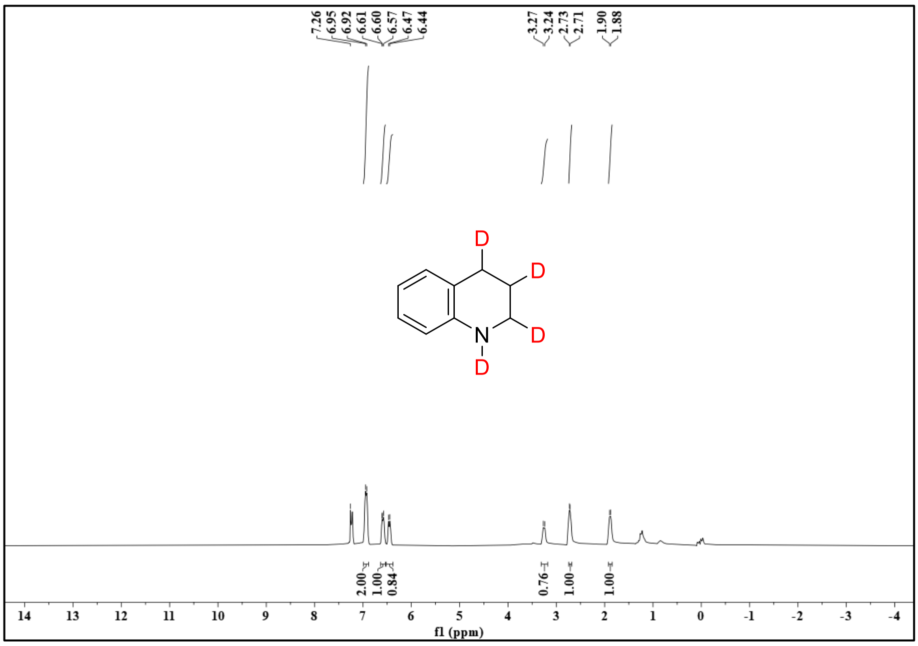

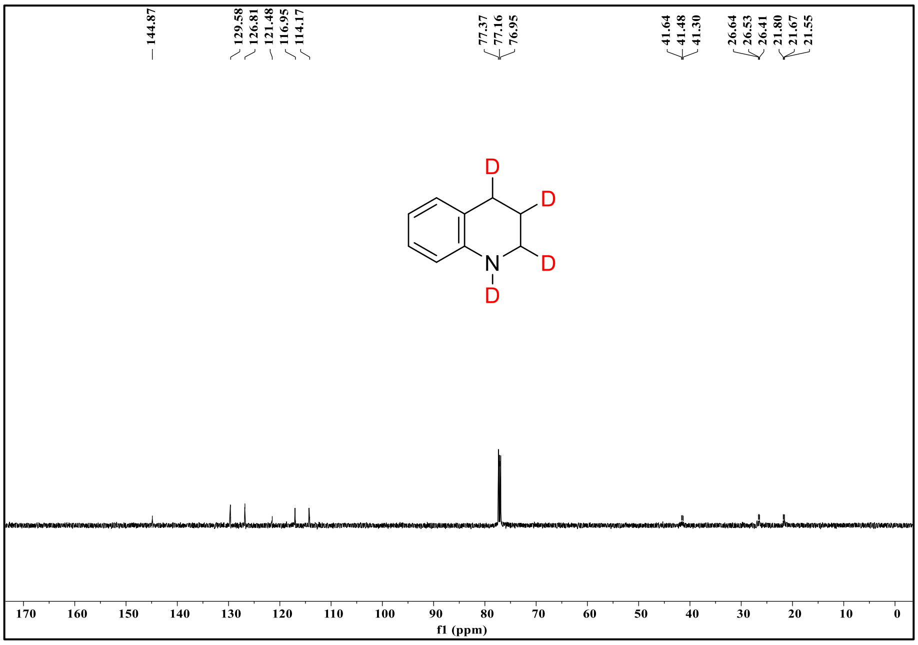

Supplementary Figure 43. **^{1}H NMR** (600 MHz, $CDCl_3$) δ [ppm] 6.93 (d, J = 14.0 Hz, 2H), 6.66 – 6.55 (m, 1H), 6.46 (d, J = 16.9 Hz, 1H), 3.25 (d, J = 17.0 Hz 1H), 2.79 – 2.68 (m, 1H), 1.89 (t, J = 12.4 Hz, 1H); **^{13}C NMR** (151 MHz, $CDCl_3$) δ [ppm] 144.87, 129.58, 126.81, 121.48, 116.95, 114.17, 41.47, 26.53, 21.67; **HR-MS** (m/z): $[M+H]^+$ calcd. for $C_9H_7D_4N$, 138.1221; found, 138.1258.

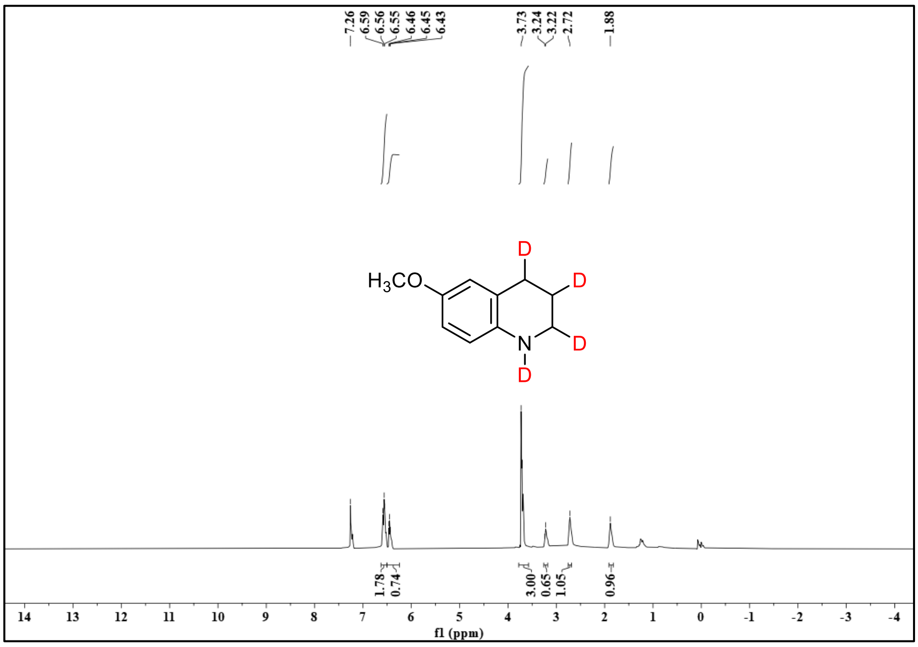

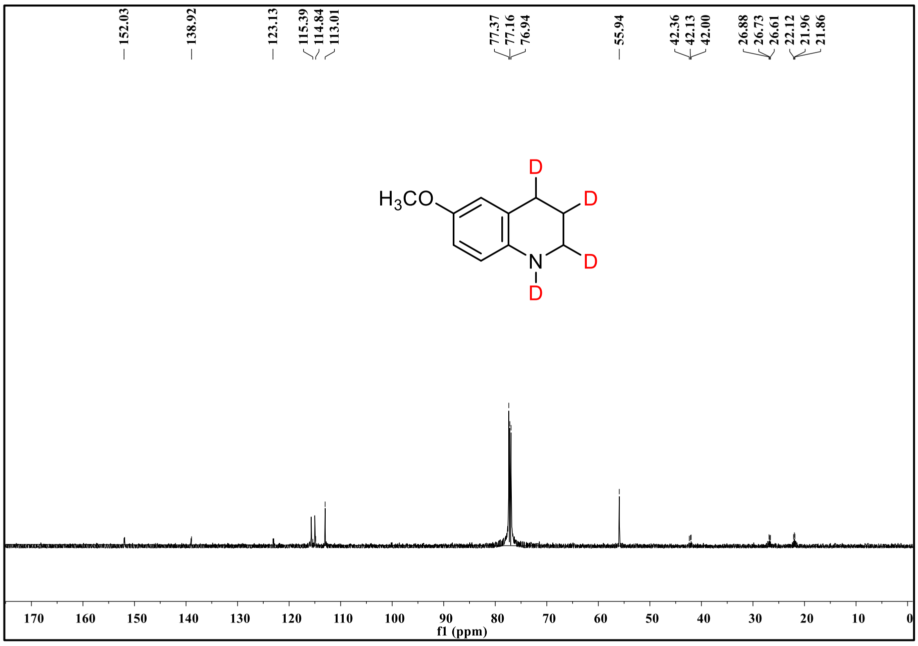

Supplementary Figure 44. **^{1}H NMR** (600 MHz, $CDCl_3$) δ [ppm] 6.62 – 6.49 (m, 2H), 6.45 (t, J = 9.3 Hz, 1H), 3.73 (s, 3H), 3.23 (d, J = 12.7 Hz, 1H), 2.72 (s, J = 8.2 Hz, 1H), 1.88 (s, 1H); **^{13}C NMR** (151 MHz, $CDCl_3$) δ [ppm] 152.03, 138.92, 123.13, 115.39, 114.84, 113.01, 55.94, 42.16, 26.74, 21.98; **HR-MS** (m/z): $[M+H]^+$ calcd. for $C_{10}H_9D_4NO$, 168.1326; found, 168.1350.

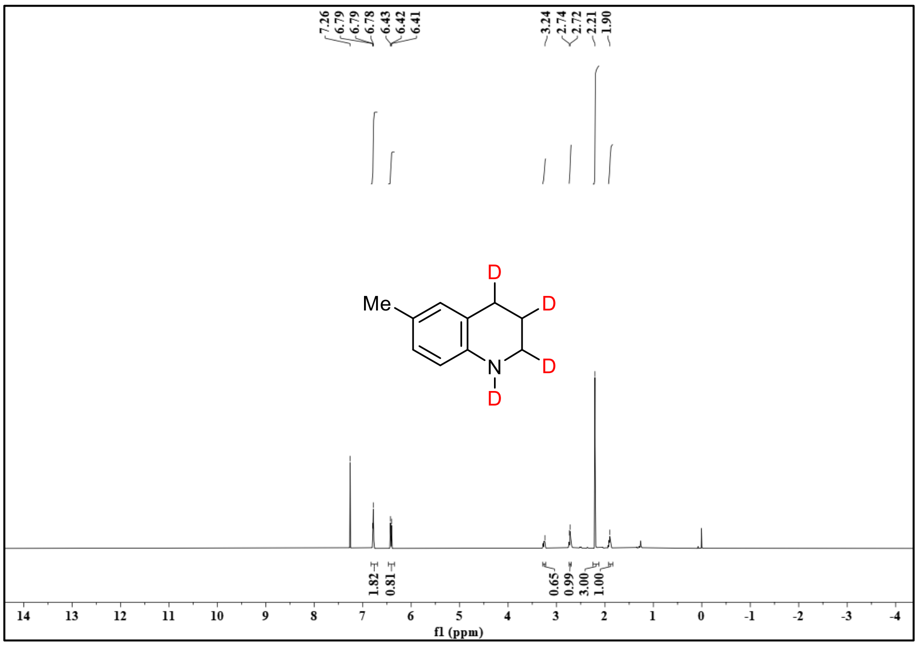

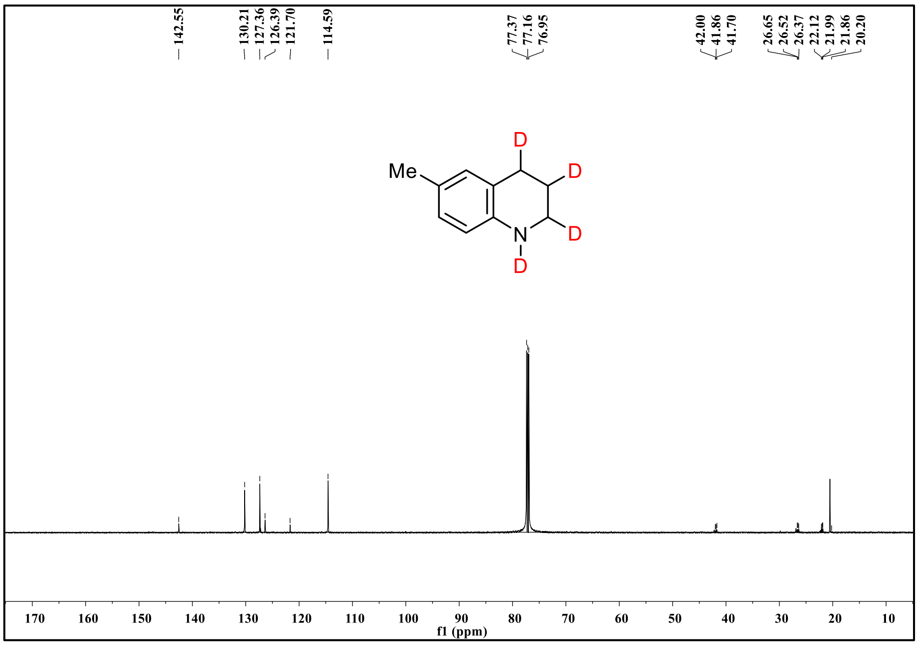

Supplementary Figure 45. ^{1}**H NMR** (400 MHz, $CDCl_3$) δ [ppm] 6.90 – 6.75 (m, 2H), 6.42 (d, J = 8.5 Hz, 1H), 3.24 (s, 1H), 2.73 (d, J = 6.6 Hz, 1H), 2.21 (s, 3H), 1.90 (s, 1H); ^{13}**C NMR** (101 MHz, $CDCl_3$) δ [ppm] 142.55, 130.21, 127.36, 126.39, 121.70, 114.59, 41.85, 26.51, 21.99, 20.20; **HR-MS** (m/z): $[M+H]^+$ calcd. for $C_{10}H_9D_4N$, 152.1377; found, 152.1397.

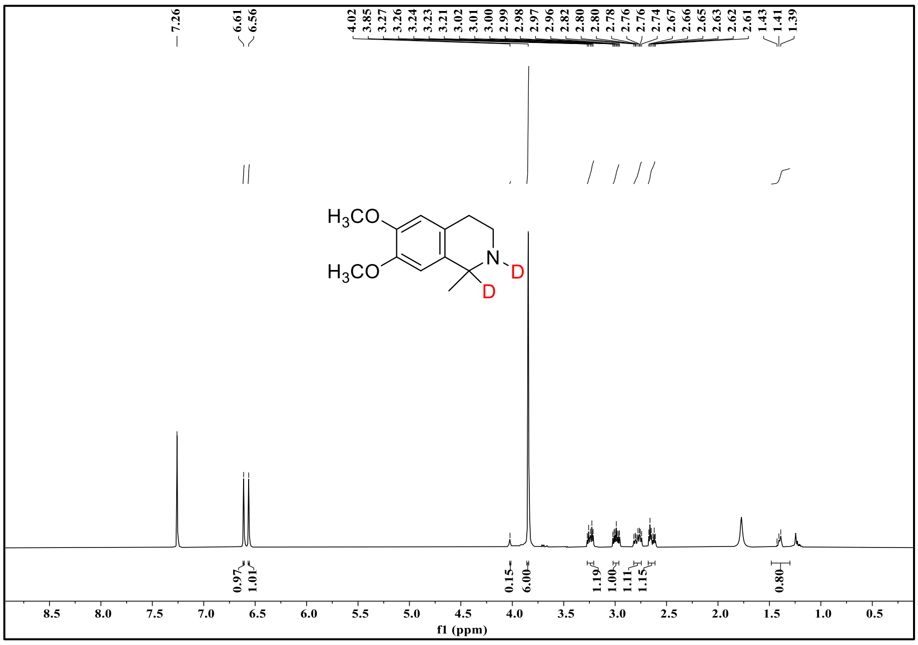

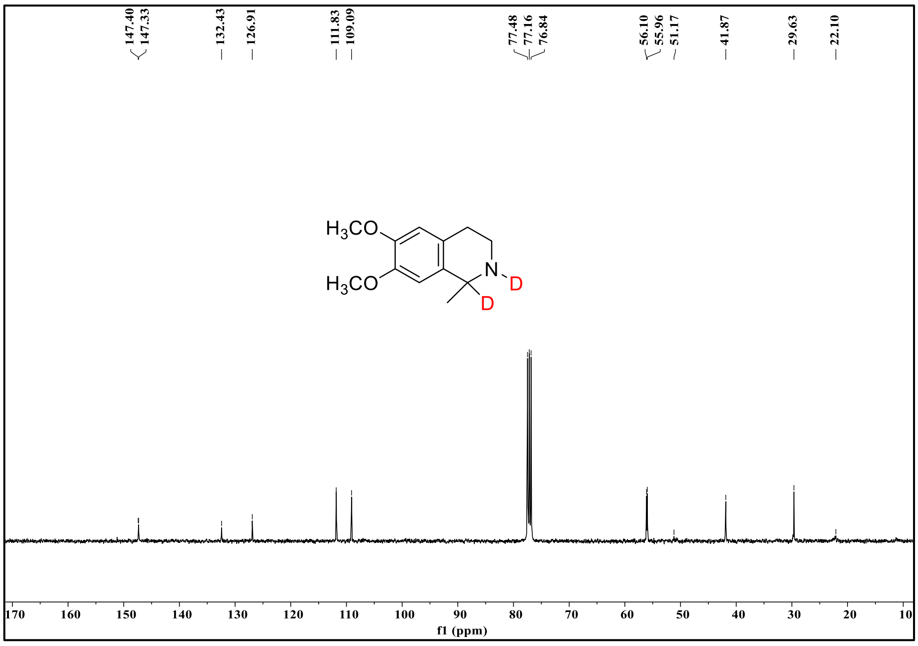

Supplementary Figure 46. ^{1}**H NMR** (400 MHz, $CDCl_3$) δ [ppm] 6.61 (s, 1H), 6.56 (s, 1H), 4.02 (s, 0.15H), 3.85 (s, 6H), 3.27 – 3.21 (m, 1H), 2.99 (ddd, J = 12.4, 8.6, 4.2 Hz, 1H), 2.82 – 2.74 (m, 1H), 2.64 (dt, J = 16.1, 4.0 Hz, 1H), 1.41 (d, J = 7.3 Hz, 3H); ^{13}**C NMR** (101 MHz, $CDCl_3$) δ [ppm] 147.40, 147.33, 132.43, 126.91, 111.83, 109.09, 56.10, 55.96, 51.17, 41.87, 29.63, 22.10; **HR-MS** (m/z): $[M+H]^+$ calcd. for $C_{12}H_{15}D_2NO_2$, 210.1463; found, 210.1549.

GC-MS spectra of 2r-2s, and 5a

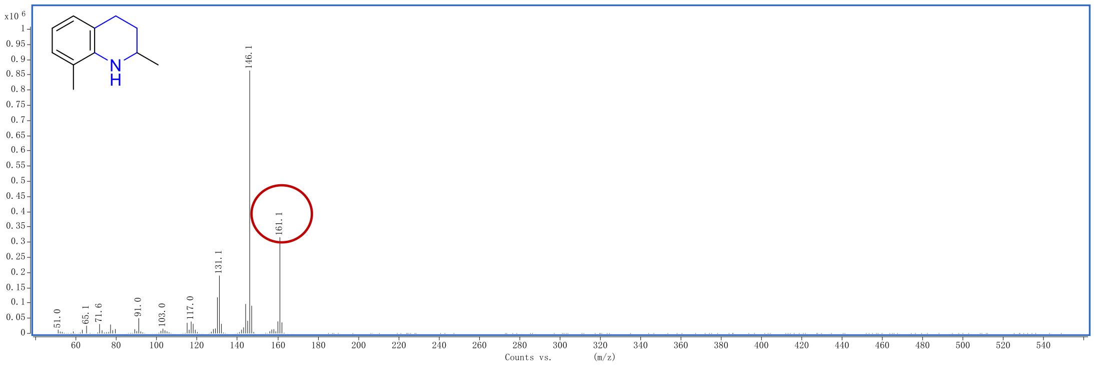

2r: **GC–MS** (EI) [M] 161.1, theoretical value for $C_{11}H_{15}N$ is 161.1.

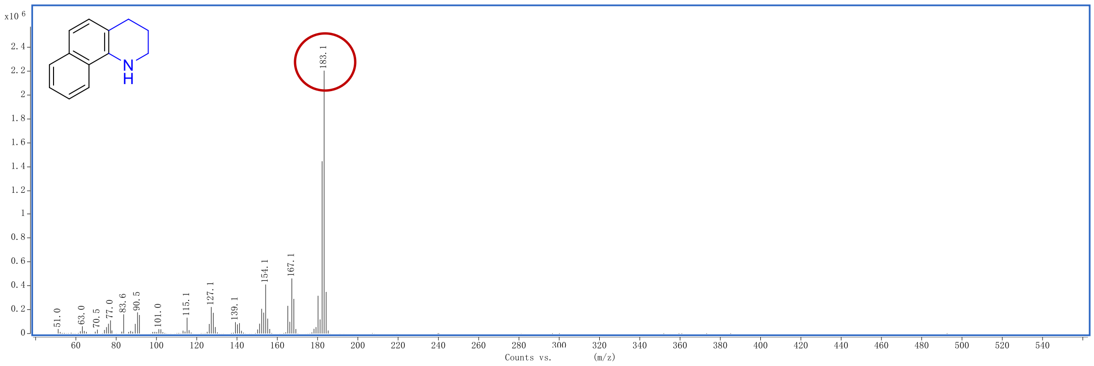

2s: **GC–MS** (EI) [M] 183.1, theoretical value for $C_{13}H_{13}N$ is 183.1.

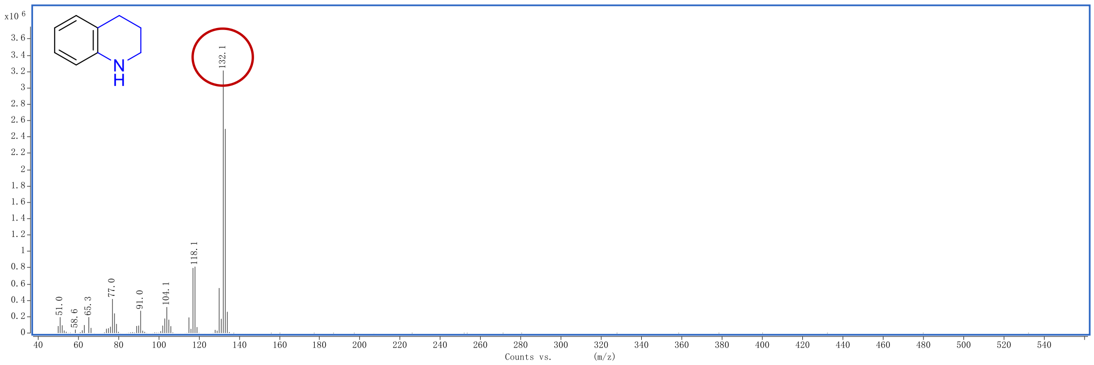

2t: **GC–MS** (EI) $[M-H]^-$ 132.1, theoretical value for $C_9H_{11}N$ is 133.1.

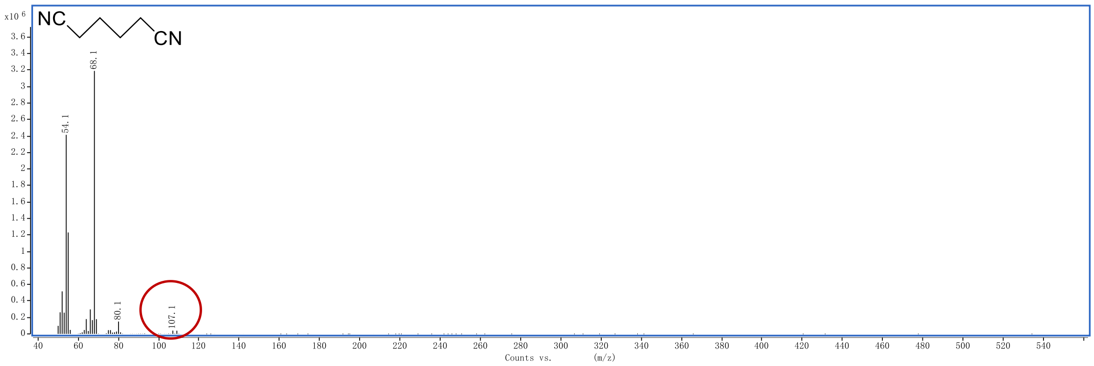

5a: **GC–MS** (EI) [M-H]$^-$ 107.1, theoretical value for $C_6H_8N_2$ [M] 108.1.

Supplementary References

1. Zhang, S. et al. Dual-active-sites design of Co@C catalysts for ultrahigh selective hydrogenation of *N*-heteroarenes. *Chem*, **6**, 2994−3006 (2020).
2. Papa, V., Cao, Y., Spannenberg, A., Junge, K. & Beller, M. Development of a practical non-noble metal catalyst for hydrogenation of *N*-heteroarenes. *Nat. Catal.* **3**, 135−142 (2020).
3. Murugesan, K., Chandrashekhar, V. G., Kreyenschulte, C., Beller, M. & Jagadeesh, R. V. A general catalyst based on cobalt core-shell nanoparticles for the hydrogenation of *N*-heteroarenes including pyridines. *Angew. Chem. Int. Ed.* **59**, 17408−17412 (2020).
4. Puche, M., Liu, L., Concepción, P., Sorribes, I. & Corma, A. Tuning the catalytic performance of cobalt nanoparticles by tungsten doping for efficient and selective hydrogenation of quinolines under mild conditions. *ACS Catal.* **11**. 8197−8210 (2021).
5. Long, X. et al. Graphitic phosphorus coordinated single Fe atoms for hydrogenative transformations. *Nat. Commun*. **11**, 4074 (2020).
6. Hervochon, J., Dorcet, V., Junge, K., Beller, M. & Fischmeister, C. Convenient synthesis of cobalt nanoparticles for the hydrogenation of quinolines in water. *Catal. Sci. Technol*. **10**, 4820−4826 (2020).
7. He, Z.-H., Li, N., Wang, K., Wang, W.-T. & Liu, Z.-T. Selective hydrogenation of quinolines over a CoCu bimetallic catalyst at low temperature. *Mol. Catal.* **470**, 120−126 (2019).
8. Gong, W. et al. Liberating N-CNTs confined highly dispersed Co-N_x sites for selective hydrogenation of quinolines. *Adv. Mater.* **31**, 1906051 (2019).
9. Han, Y. et al. Ordered porous nitrogen-doped carbon matrix with atomically dispersed cobalt sites as an efficient catalyst for dehydrogenation and transfer hydrogenation of N-heterocycles. *Angew. Chem. Int. Ed.* **57**, 11262−11266 (2018).
10. Wei, Z. et al. Cobalt encapsulated in N-doped graphene layers: an efficient and stable catalyst for hydrogenation of quinoline compounds. *ACS Catal.* **6**, 5816−5822 (2016).
11. Chen, F. et al. Selective catalytic hydrogenation of heteroarenes with *N*-graphene-modified cobalt nanoparticles (Co_3O_4-Co/NGr@α-Al_2O_3). *J. Am. Chem. Soc*. **137**, 11718−11724 (2015).
12. Lu, L., Li, H., Zheng, Y., Bu, F. & Lei A. Facile and economical electrochemical dehalogenative deuteration of (hetero)aryl halides. *CCS Chem*. **2**, 2669−2675 (2020).
13. Wang, X. et al. General and practical potassium methoxide/disilane-mediated dehalogenative deuteration of (hetero)arylhalides. *J. Am. Chem.Soc.* **140**, 10970−10974 (2018).
14. Loh, Y. et al. Photoredox-catalyzed deuteration and tritiation of pharmaceutical compounds. *Science* **358**, 1182−1187 (2017).
15. Yu, R., Hesk, D., Rivera, N., Pelczer, I. & Chirik, P. J. Iron-catalysed tritiation of pharmaceuticals. *Nature* **529**, 195−199 (2016).
